# Ethylene and Auxin: Hormonal Regulation of Volatile Compound Production During Tomato (*Solanum lycopersicum* L.) Fruit Ripening

**DOI:** 10.3389/fpls.2021.765897

**Published:** 2021-12-10

**Authors:** Eric de Castro Tobaruela, Bruna Lima Gomes, Vanessa Caroline de Barros Bonato, Elis Silva de Lima, Luciano Freschi, Eduardo Purgatto

**Affiliations:** ^1^Department of Food and Experimental Nutrition, School of Pharmaceutical Sciences, University of São Paulo (USP), São Paulo, Brazil; ^2^Food Research Center (FoRC), São Paulo, Brazil; ^3^Department of Botany, Institute of Bioscience, University of São Paulo (USP), São Paulo, Brazil

**Keywords:** climacteric fruit, crosstalk, carotenoids, aromatic volatiles, fruit quality

## Abstract

As the auxin-ethylene interaction in climacteric fruit ripening has been highlighted, the hormonal regulation of aroma changes in climacteric fruits requires clarification. The influence of both phytohormones on the volatile organic compound (VOC) metabolism was evaluated during tomato (*Solanum lycopersicum* L.) fruit ripening. Tomato fruits cv. Micro-Tom and Sweet Grape at the mature green stage were randomly grouped according to treatment with ethylene (ETHY), auxin (IAA), or both (ETHY + IAA). At middle ripening, Micro-Tom ETHY + IAA fruits present VOC profiles similar to those of ETHY fruits, while Sweet Grape presents VOC profiles closer to those of IAA fruits. At full ripeness, Micro-Tom and Sweet Grape ETHY + IAA fruits show profiles closer to those of IAA fruits, suggesting that the auxin overlaps the ethylene effects. Aroma compounds positively correlated with consumer preferences (2-isobutylthiazole, 6-methyl-5-hepten-2-one, and others) are identified in both cultivars and have their contents affected by both hormone treatments. The transcription of genes related to the biosynthesis of important tomato VOCs that have fatty-acid and carotenoid precursors evidences their regulation by both plant hormones. Additionally, the results indicate that the observed effects on the VOC metabolism are not restricted to the Micro-Tom cultivar, as these are also observed in the Sweet Grape cultivar. In conclusion, ethylene and auxin directly regulate the metabolic pathways related to VOC formation, impacting tomato aroma formation during ripening since Micro-Tom fruits apparently at the same maturation stage have different aromas.

## Introduction

Tomatoes (*Solanum lycopersicum* L.) are among the most cultivated and consumed horticultural crops worldwide^1^. They have been extensively studied as a reference for climacteric fruits and a model system for fleshy fruit development and ripening due to their advantages over other species of agronomic interest (Gapper et al., 2013; Zhu et al., 2018; Food and Agriculture Organization Corporate Statistical Database [FAOSTAT], 2021). In these fruits, ripening is associated with a rapid spike in the biosynthesis of ethylene, a phytohormone that plays a critical role in controlling ripening (Tucker et al., 2017). During this process, climacteric fruits experience an initial auto-inhibitory phase (system 1) followed by a rapid increase in ethylene emission during the initiation of an autocatalytic phase (system 2; Fenn and Giovannoni, 2021). Without undermining the role of ethylene in fruit ripening, new perspectives have been introduced on ripening regulation that indicate coordinated action between ethylene and other phytohormones. Auxin was shown to significantly affect the interplay between other plant hormones, and it is now commonly accepted as a main regulator of fruit ripening (Muday et al., 2012; Kumar et al., 2014). Indole-3-acetic acid is the main auxin in plants (Korasick et al., 2013). Although the intricate mechanisms underlying auxin regulation remain largely unclear, fruit ripening studies have shown the roles of a few auxin-related genes in controlling fruit firmness (Guillon et al., 2008) and regulating pigment accumulation (Breitel et al., 2016; Hao et al., 2016). Furthermore, evidence suggests crosstalk between ethylene and auxin during ripening, with auxin having the opposite effect to that of ethylene (Su et al., 2015; Li et al., 2016). Elevated auxin levels delay the fruit ripening process, and their biosynthesis decreases with the transition to fruit ripening (Kumar et al., 2014). Auxin is crucial for triggering ripening and impacts the transition between the two ethylene production systems. Therefore, it does not necessarily inhibit fruit ripening (Ross et al., 2011; McAtee et al., 2013; Seymour et al., 2013).

The interplay between multiple phytohormones regulating the metabolic pathways involved in fruit ripening can occur at different levels, including through interaction between components of phytohormone signal transduction (Muday et al., 2012; Li et al., 2021). Regarding ethylene-auxin crosstalk, recent reports have elucidated antagonistic effects of the two plant hormones, including auxin acting as a ripening repressor and thereby opposing the known role of ethylene in inducing ripening in tomato fruit (Su et al., 2015; Li et al., 2016). However, since the auxin-ethylene relationship is extremely intricate, some of the phenotypic effects have yet to be assigned to either phytohormone (Wang et al., 2021).

Tomato aroma quality is strictly ripening-dependent once most of the volatile organic compounds (VOCs) are produced from fatty acids, carotenoids, and amino acids in metabolic processes that occur during ripening process. These compounds accumulate at the onset of tomato ripening and peak at either full ripening or shortly before, producing the tomato fruit aroma (Klee and Giovannoni, 2011; Karlova et al., 2014). In conjunction with hormonal factors, the VOC profile is also affected by cultivation conditions, ripening stage, and postharvest treatment (Baldwin et al., 2015; Lee et al., 2018; Zhao et al., 2019). Another increasingly studied aspect is the wide range of phenotypic variation and metabolic diversity of tomato cultivars. Several hundred VOCs were identified in the fruits of nearly 400 tomato cultivars. However, only 23 of these were identified as being responsible for the characteristic aroma of tomatoes, while 13 were found to be important in terms of consumer acceptance: methional; 3-methylbutanenitrile; 2-methyl-1-butanol; 3-methyl-1-butanol; 2-isobutylthiazole; 1-octen-3-one; β-ionone; 3-methylbutanoic acid; (E,E)-2,4-decadienal; 3-methylbutanal; (E)-2-heptenal; and 6-methyl-5-hepten-2-one (Tieman et al., 2017). Comparing the flavors of 71 tomato accessions in China, another study highlighted 15 VOCs as compounds that could be used as parameters for evaluating tomato flavor (Cheng et al., 2020). Evidencing this diversity in tomato aroma, Tieman et al. (2017) also identified some flavorful components that have been lost with breeding. Other recent studies have revealed substantial gene loss and intense negative selection of genes and promoters during tomato domestication and improvement. Gao et al. (2019) identified a rare allele of *TomLoxC* that may have undergone negative selection during domestication. This tomato lipoxygenase gene is involved in apocarotenoid production, which contributes to the desirable tomato aroma.

Few studies have evaluated the effects of phytohormone crosstalk on ripening parameters. Knowledge of aroma regulation is limited, and the role of ethylene and auxin in aroma formation during climacteric fruit ripening remains almost unknown. According to Fenn and Giovannoni (2021), VOC accumulation and the role of plant hormones in VOC production are intriguing and currently expanding areas of inquiry that are open to further exploration. In this context, this study explored the interactions between ethylene and auxin, as well as the role of each phytohormone in the VOC metabolism in two tomato cultivars. We explored the effects of exogenous ethylene and auxin treatments on the VOC profiles and the expression of key genes related to regulating the metabolism of tomato aroma compounds. Finally, our results provide insights into the contributions of ethylene, auxin, and their interactions to regulating aroma metabolism during tomato fruit ripening.

## Materials and Methods

### Plant Materials and Phytohormone Treatments

Tomato plants (*S. lycopersicum* cv. Micro-Tom and Sweet Grape) were grown under standard greenhouse conditions. Fruit samples were harvested at the mature green stage and randomly separated into four groups (*n* = 100 fruits by group) according to phytohormone treatments: CTRL (without treatment), ETHY (ethylene treatment), IAA (indole-3-acetic acid treatment), and ETHY + IAA (both treatments). After harvested, tomato fruits were sterilization with 0.1% aqueous sodium hypochlorite solution for 15 min and hormonal treatments were immediately applied. During the experiments, fruits were left to ripen spontaneously in a 323 L chamber at 22°C, for a 16-h-day/8-h-night cycle at 80% relative humidity. Ethylene treatments were performed using a gaseous hormone at 10 μL.L^–1^ for 12 h. The indole-3-acetic acid solutions were prepared at 100 μM in 10 mM MES buffer at pH 5.6 using 3% sorbitol and injected through the calyx end as described by Su et al. (2015). Fruits from the IAA group received indole-3-acetic acid solution, while fruits from the ETHY + IAA group were exposed to gaseous ethylene before being infiltrated with auxin solution. Fruits from the ETHY and CTRL groups were injected with a buffer solution only to maintain a consistent injection method for all samples. Both treatments were optimized under our laboratory conditions and the phytohormone concentrations were selected as they were sufficient to change tomato ripening. Two experimental blocks were performed with Micro-Tom fruits, while one was performed with Sweet Grape fruits. In the first Micro-Tom and Sweet Grape experiments, fruits in all groups were frozen during the ripening stage of the CTRL group. The second Micro-Tom experiment had a different experimental design, with samples being frozen when their group reached the defined ripening stages. During all the experiments, the ethylene emission and peel color shift were evaluated daily as ripening parameters. For further analyses, at least five fruits from each group were collected at different points, covering three ripening stages: mature green, breaker, and red. Samples were then frozen in liquid nitrogen and stored at −80°C.

### Ethylene Emission

Five fruits from each group were individually placed in airtight glass containers and left at 25°C. After 1 h, 1 mL samples were collected from the headspace with a gas-tight syringe through a rubber septum for ethylene analysis. This analysis was performed by gas chromatography (Agilent Technologies Inc., Santa Clara, United States, model HP-6890). A flame ionization detector was employed, and an HP-Plot Q column (30 m × 0.53 mm × 40 μm, Agilent Technologies) was used. The injector and detector temperatures were set at 250°C, and the oven temperature was set at 30°C. The injections were performed in pulsed splitless mode. Helium was used as the carrier gas (1 mL.min^–1^).

### Peel Color Characterization

The peel color was measured using a HunterLab ColorQuest XE instrument (Hunter Associates Laboratories) in terms of L (lightness), A (redness and greenness), and B (yellowness and blueness). The hue value was used to represent color variations and was defined as described by Fabi et al. (2007). At least five representative measurements were taken from each experimental group.

### Extraction of Volatile Organic Compounds and Solid-Phase Microextraction–GC–MS Analysis

The headspace volatile production of tomato fruit was determined by solid-phase microextraction (SPME). A pool of five fresh fruits was homogenized with 30% sodium chloride solution (Merck). Aliquots of 10 g were placed in vials and frozen at −20°C. Samples were thawed under agitation at 40°C immediately before analysis. The headspace equilibrium time was 10 min, and the adsorption time was 50 min. The SPME fiber (PDMS/DVB/CAR 50 μm, Supelco Co.) was injected directly into an HP-6890 gas chromatograph (Agilent Technologies) coupled to a mass selective detector (HP-5973, Agilent Technologies) and held for 10 min for desorption of VOCs. The injector temperature was 200°C. Components were then separated using a Supelcowax 10 capillary column (30 m × 0.25 mm × 0.25 μm), and the oven temperature was programmed to increase from 40 to 150°C at 2°C.min^–1^. These conditions were previously optimized and selected according to the higher number of peaks and total area of the chromatogram. VOCs were identified by comparison using the NIST (NIST98, version 2.0, Gaithersburg, United States) and confirmed with the spectral data available from the MassBank of North America (MoNA^2^). Identification was also based on comparing the retention indices (RIs) of a series of *n*-alkanes (C7–C30, Supelco) with those reported in the literature and the retention times and mass spectra with those of authentic external standards. A pool of the following volatile compound external standards (Sigma–Aldrich) was prepared for use in identifying the compounds by mass spectral comparison: (E)-2-hexenal, hexanal, 1-hexanol, (Z)-3-hexen-1-ol, (Z)-3-hexenal, 1-penten-3-one, 6-methyl-5-hepten-2-one, guaiacol, 1-pentanol, pentanal, geranyl acetone, β-ionone, citral, methyl salicylate, 2-isobutylthiazole, 2-phenylethanol, and (E)-2-octenal. All analyses were performed in triplicate.

### Carotenoid Analysis

Representative samples for each experimental group were used for carotenoid extractions. Five frozen fruits were powdered, and analyses were performed according to the method developed by Sérino et al. (2009).

### RNA Isolation and Quantitative RT-PCR Analysis

Gene expression analyses were performed according to Fabi et al. (2012), following the “Minimum Information for Publication of Quantitative Real-Time PCR Experiments – MIQE” (Bustin et al., 2009). All the primers used for amplification are listed in Supplementary Table 1.

The total RNAs from five individual fruits at each experimental stage were extracted using Concert™ Plant RNA Reagent (Invitrogen™) and treated with the “Ambion^®^ DNA-free™ DNase Treatment and Removal Reagents” kit (Invitrogen™) for genomic DNA removal. RNAs were verified by agarose gel electrophoresis. For the cDNA synthesis, 1 μg of RNAs, measured spectrophotometrically, was used in reverse transcription reactions according to the instructions for the “ImProm-II™ Reverse Transcription System” kit (Promega). In total, 10 μL of real-time PCR reactions were set using the “Power SYBR Green Master Mix” kit (Applied Biosystems). Reactions were run in a QuantStudio 7 Flex system (Applied Biosystems) programmed to remain at 95°C for 5 min, 60 cycles of 95°C for 15 s, 60°C for 30 s, and 72°C for 30 s. The TIP41-like protein (TIP) and expressed unknown protein (EXP) genes were used as the internal controls, and the relative expressions were calculated according to the method developed by Pfaffl (2001).

### Statistical Analysis

SPSS Version 19.0 (SPSS Inc., Chicago, IL, United States) was used to perform statistical analysis. Data were analyzed using Student’s *T*-tests or a one-way ANOVA with a subsequent Tukey’s test to evaluate the effects of phytohormone treatments on VOCs and other evaluated parameters at different ripening stages. Differences were considered significant at *p* < 0.05. VOC data were uploaded to MetaboAnalyst 5.0 (Pang et al., 2021) for a heatmap, hierarchical clustering analysis (HCA), partial least squares discriminant analysis (PLS-DA), and variable importance in projection (VIP) score after normalization by median, log transformation, and Pareto scaling. Linear regression analysis was applied to gene expression during fruit ripening, with the phytohormone treatment data being used as predictor variables (*x*) and transcript levels as response variables (*y*) and assuming a significance level of *p* < 0.05.

## Results

### Overview of Ethylene and Auxin Effects on Primary Ripening Parameters

**FIGURE 1:**
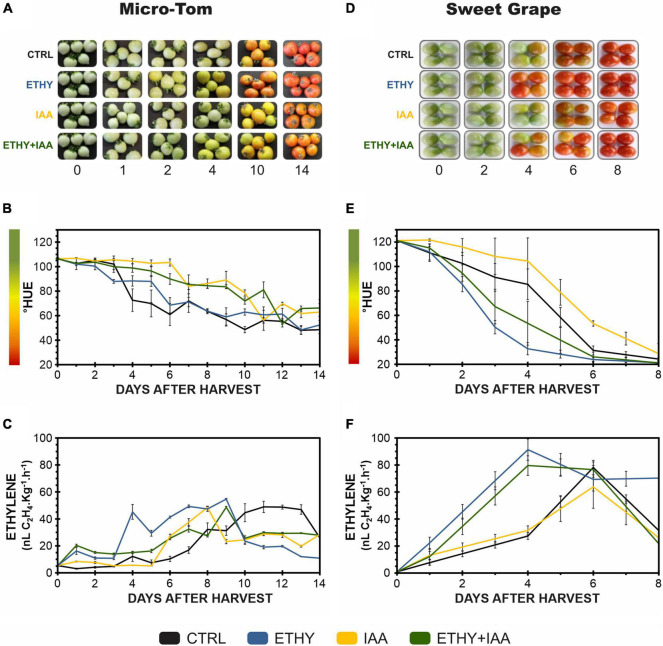
Effects of ethylene, auxin, and both treatments on fruit color and ethylene emission during tomato (*Solanum lycopersicum* L.) cv. Micro-Tom **(A–C)** and Sweet Grape **(D–F)** fruit ripening. **(A,D)** Illustrative photographs of tomato fruits. **(B,E)** Peel color expressed by hue angle. **(C,F)** Ethylene emission. CTRL, control group. ETHY, ethylene-treated group. IAA, indole-3-acetic acid-treated group. ETHY + IAA, group treated with both hormones. Vertical bars represent the standard deviation of three replicates (*n* = 3).

The ripening of tomato (*S. lycopersicum* L.) fruits treated with one phytohormone (ETHY or IAA) or a combination thereof (ETHY + IAA) was monitored daily using peel color characterization and ethylene emission as primary ripening parameters (Figure 1). For the first Micro-Tom and Sweet Grape experiments, CTRL fruits (without treatment) were used as reference of ripening stage, what means that fruits of all groups were frozen according to the ripening stage of the CTRL. Photographs of Micro-Tom and Sweet Grape fruits during ripening are presented in Figures 1A,D, respectively. As expected, Micro-Tom fruits showed a color shift (Figure 1B), and their ethylene emission (Figure 1C) was accelerated by ethylene treatment. ETHY fruits showed ethylene emission approximately four times higher than that of CTRL fruits on the 1st day after harvest (DAH) and showed higher concentrations between 2 and 4 DAH. The IAA and ETHY + IAA groups showed delays at the beginning of the color shift; however, the climacteric peak was advanced compared with that of the CTRL group. Fruits treated with both plant hormones showed less variation in ethylene levels over the evaluated ripening days. On the 14th DAH, CTRL and ETHY fruits had reached full ripeness, while auxin-treated fruits (IAA and ETHY + IAA) did not show the observed red color. Sweet Grape fruits presented the same response profiles when subjected to the phytohormone treatments. As observed in the Micro-Tom experiment, the color shift (Figure 1E) and ethylene emission (Figure 1F) were accelerated by ethylene treatment and delayed by auxin. However, Sweet Grape tomatoes treated with both hormones showed results closer to those of ETHY than of IAA fruits. On the 6th DAH, ETHY fruits had reached full ripeness, while the same stage was reached on the 8th DAH for the CTRL and ETHY + IAA fruits. Those treated only with auxin did not show the observed red color.

### Volatile Organic Compound Profiles of Micro-Tom and Sweet Grape Fruits Differ During Ripening

**FIGURE 2:**
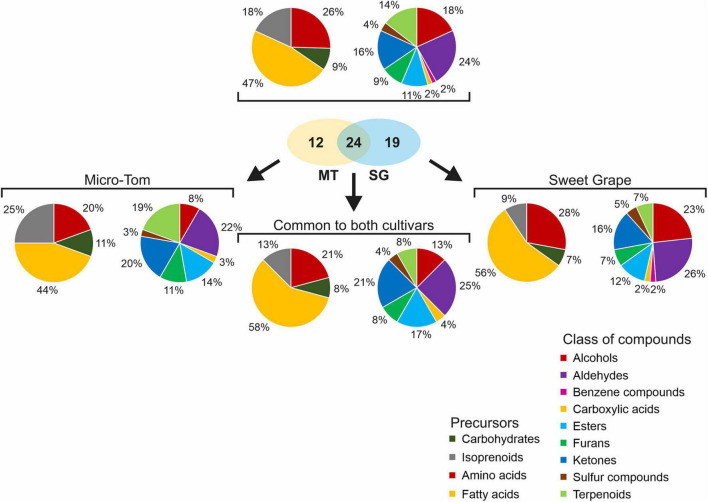
Relative composition of volatile organic compound (VOC) profiles of tomato (*S. lycopersicum* L.) fruits cv. Micro-Tom and Sweet Grape, as well as the “global” and the “common” VOC profiles. Left pie charts show the relative composition according to VOC precursors while right pie charts show the relative composition according to their compounds classes.

In total, 55 VOCs were identified as contributing to the tomato aroma, of which 24 were common to both cultivars (Figure 2). These compounds were mainly aldehydes (13), alcohols (10), ketones (9), and terpenoids (8); however, esters (6), furans (5), sulfur compounds (2), a benzene compound (1), and a carboxylic acid (1) were also found. The VOC profile of Sweet Grape fruits was more similar to the profile common to both cultivars than that of Micro-Tom, based on the compound classes. Both cultivars had many aldehydes (22% of the VOC profile in Micro-Tom and 26% in Sweet Grape); however, Sweet Grape fruits presented a profile rich in alcohols (23%) while Micro-Tom fruits had a profile with higher ketone (20%) and terpenoid contents (18%). Regarding the metabolic precursors, 26 compounds had fatty-acid precursors, 14 were obtained through amino-acid degradation, 10 were directly derived from isoprenoids, and five were derived from carbohydrates. Approximately 56% of the compounds comprising the VOC profile of Sweet Grape fruits were derived from fatty acids, making this profile more similar to that common to both cultivars (58%). Micro-Tom fruits had almost three times more isoprenoid-derived compounds than Sweet Grape. Moreover, only 44% of their VOC profile was derived from fatty acids.

**FIGURE 3:**
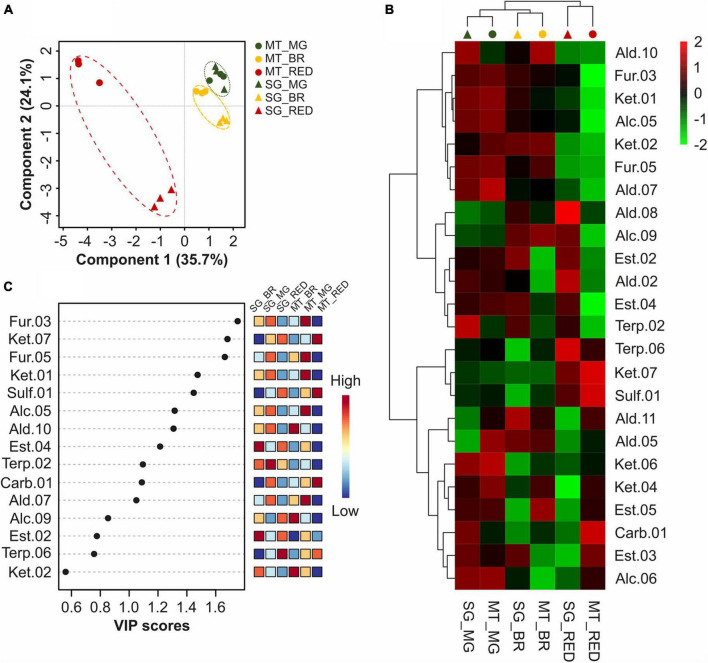
Comparison of VOC profiles of tomato (*S. lycopersicum* L.) fruits cv. Micro-Tom and Sweet Grape at mature green, breaker, and red stages. **(A)** Partial least squares discriminant analysis (PLS-DA). **(B)** Heatmap with hierarchical clustering. **(C)** Variable importance in projection (VIP) score plot for the TOP15 most important VOCs identified by the PLS-DA. MT, Micro-Tom. SG, sweet grape. MG, mature green stage. BR, breaker stage. RED, red stage. Each value is presented as the mean (*n* = 3).

Multivariate analyses were performed to differentiate the two cultivars’ fruits at the mature green, breaker, and red stages based on their VOC profiles to understand the changes in the VOC profile during ripening and identify the similarities and differences between untreated Micro-Tom and Sweet Grape fruits (Figure 3). The PLS-DA showed that the two cultivars were undiscriminated at the mature green stage, differed at the breaker stage, and were clearly discriminated at the red stage (Figure 3A), with compounds of different classes contributing significantly to explaining the PLS-DA model (Figure 3B). Furans (2-ethylfuran and 2-propylfuran) and ketones (6-methyl-5-hepten-2-one and 3-pentanone), in addition to (Z)-3-hexen-1-ol and 2-isobutylthiazole, were the most important compounds in distinguishing the ripening stages in the two cultivars. The heatmap in Figure 3C indicates that the VOC profiles of both cultivars are more similar at the mature green and breaker stages than at the red stage, besides the existence of four VOC clusters. This indicated that, although specific compounds presented significant differences at the mature green stage, the cultivars presented more similar VOC profiles at the beginning than at the end, the latter being responsible for the final specific aroma of each cultivar.

### Hormonal Regulation of Volatile Organic Compound Profiles During Tomato Ripening

Volatile organic compound profiles were analyzed by SPME–GC–MS at the mature green, breaker, and red stages to investigate how each hormonal treatment, and their combination, affected the tomato fruit aroma. Additionally, these were analyzed the 1st day after the treatments (01 DAH). In total, 62 VOCs were identified and confirmed in Micro-Tom fruits, of which 36 contributed to the aroma through odor notes and varying odor thresholds (Supplementary Table 2). The number and relative abundance (%) of compounds changed during ripening, in addition to being affected by the hormonal treatments (Supplementary Figure 1). These compounds were mainly aldehydes (8), ketones (7), and terpenoids (7); however, esters (5), furans (4), alcohols (3), a carboxylic acid (1), and a sulfur compound (1) were also found (Supplementary Figure 1A). Regarding the metabolic precursors, sixteen compounds had fatty-acid precursors, nine were directly derived from isoprenoids, seven were obtained through amino-acid degradation, and four were derived from carbohydrates (Supplementary Figure 1B).

**FIGURE 4:**
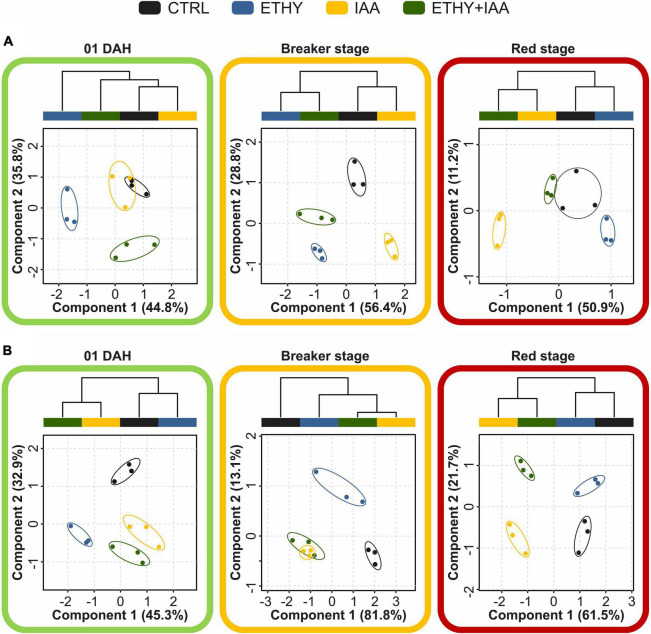
Effects of ethylene, auxin, and both treatments on VOC profiles during tomato (*S. lycopersicum* L.) fruits cv. Micro-Tom and Sweet Grape ripening. **(A)** Partial least squares discriminant analysis (PLS-DA) and hierarchical clustering analysis (HCA) of VOCs identified in Micro-Tom fruits at 01, 04 (breaker), and 14 (red) days after harvest (DAH). **(B)** PLS-DA and HCA of VOCs identified in Sweet Grape fruits at 01, 04 (breaker), and 08 DAH (red).

Multivariate analyses (PLS-DA, HCA, and VIP score) were performed to obtain an overall description of the effects of the plant hormones on VOC profiles at 01, 04, and 14 DAH. The PLS-DA results are shown in Figure 4A and Supplementary Figure 2A. At the beginning of ripening, Micro-Tom fruits presented VOC profiles comprising 28 of the 36 previously selected compounds, and 1 of these presented abundances with significant differences (*p* < 0.05) between at least two groups. PLS-DA explained 80.6% (component 1, 44.8%; component 2, 35.8%) of the total variance between the CTRL and treated samples. Volatile profile differences between ETHY and the other groups were evidenced primarily by component 1, while component 2 separated ETHY + IAA from the other groups. HCA reinforced differences visualized by PLS-DA, showing that the IAA and CTRL groups presented similar VOC profiles, with both being more similar to the ETHY + IAA than to the ETHY group. Based on PLS-DA, aldehydes, alcohols, and ketones were the most important in distinguishing the CTRL and treated samples. Four of the eight aldehydes found at the beginning of ripening [hexanal, 3-hexenal, (E)-2-hexenal, and (E)-2-octenal] presented abundances with significant differences (*p* < 0.05) between at least two groups. These compounds had fatty-acid precursors and were found with higher contents in the CTRL and ETHY + IAA fruits, derived from fatty acids, found with higher contents in the CTRL and ETHY fruits. Concerning the ketones, 2-heptanone and 1-hepten-3-one contents were higher in treated fruits and with significant difference between ETHY and CTRL groups.

After 3 DAH, when the CTRL fruits reached the breaker stage, their VOC profiles comprised 31 compounds, 26 of which presented abundances with significant differences (*p* < 0.05) between at least two groups. PLS-DA explained 85.2% of the total variation between samples. Component 1 (56.4%) separated the CTRL and IAA from ETHY and ETHY + IAA groups, and component 2 (28.8%) separated the ETHY and IAA groups from the CTRL and ETHY + IAA groups. In addition to PLS-DA, HCA showed that the ETHY and ETHY + IAA groups had more similar VOC profiles, as did the IAA and CTRL groups. Hexanal, (E)-2-hexenal, (E)-2-heptenal, 6-methyl-5-hepten-2-one, and 2-isobutylthiazole, aroma compounds important to consumer acceptance, were found with higher contents in ETHY and ETHY + IAA fruits. Other important VOCs, including 2-ethylhexan-1-ol and (E,E)-2,4-hexadienal, were found only in the CTRL fruits, while decanal and β-ionone were exclusive to ETHY fruits.

When the CTRL group was at the red stage, the VOC profiles comprised 27 compounds, only 11 of which presented abundances with significant differences (*p* < 0.05) between at least two groups. PLS-DA explained 62.1% of the total variation between samples. Component 1 (50.9%) separated the IAA from the other groups, while component 2 (11.2%) separated the IAA and ETHY from the CTRL and ETHY + IAA groups. Additionally, HCA showed that the IAA and ETHY + IAA fruits presented more similar VOC profiles, while ETHY presented profile more similar to the CTRL group. Regarding fatty-acid-derived volatiles, the accumulation of (Z)-3-hexen-1-ol, 2-propenal, hexanal, (E)-2-hexenal, (E)-2-octenal, 1-penten-3-one, and 1-hepten-3-one was affected by exogenous ethylene and auxin treatment during ripening. Hexanal and 2-propenal were found in higher contents in IAA and ETHY + IAA than in ETHY fruits. However, 1-penten-3-one, 1-hepten-3-one, (Z)-3-hexen-1-ol, and (E)-2-octenal were fatty-acid-derived volatiles with higher contents in ETHY than in IAA and ETHY + IAA fruits. Hexanal, (E)-2-hexenal, and (Z)-3-hexen-1-ol were fatty-acid-related volatiles found with higher contents in the tomato aroma profiles, at 10, three, and two times higher than the other VOCs, respectively. Other compounds, including citral, 6-methyl-5-hepten-2-one, and methyl salicylate, were found with higher contents in ETHY and ETHY + IAA than in IAA fruits (Supplementary Figure 3).

Knowing that Micro-Tom and Sweet Grape fruits are highly similar at the onset of ripening but different when fully ripe, it was possible to identify the effects of the same hormonal treatments on Sweet Grape fruits and compare their results with the effects on Micro-Tom fruits. In total, 51 compounds were identified and confirmed in Sweet Grape fruits, of which 43 had the odoriferous note described in the literature (Supplementary Table 3). As observed in the Micro-Tom experiment, the number and the relative abundance (%) of compounds were affected by the ripening stage and hormonal treatment (Supplementary Figure 4). These compounds were mainly aldehydes (11), alcohols (10), and ketones (7); however, esters (5), terpenoids (3), furans (3), sulfur compounds (2), a benzene compound (1), and a carboxylic acid (1) were also found (Supplementary Figure 4A). Regarding the metabolic precursors, 24 compounds had fatty-acid precursors, 12 were obtained through amino-acid degradation, four were directly derived from isoprenoids, and three were derived from carbohydrates (Supplementary Figure 4B).

On the 1st DAH (Figure 4B and Supplementary Figure 2B), Sweet Grape fruits presented VOC profiles comprising 28 of the 43 previously selected compounds, only 10 of which presented abundances with significant differences (*p* < 0.05) between at least two groups. PLS-DA explained 78.2% (component 1, 45.3%; component 2, 32.9%) of the total variance between the CTRL and treated samples. Component 1 separated the ETHY from the other groups, while the VOC profile of the CTRL group was separated by component 2. HCA reinforced the differences visualized by PLS-DA, where the CTRL and ETHY groups presented more similar VOC profiles, as did the IAA and ETHY + IAA groups. At this stage, some important fatty-acid-derived volatiles, including 3-pentanone, hexanal, and (E)-2-hexenal, were affected by the hormone treatments and showed higher contents in the CTRL than in treated fruits. Other fatty-acid derivatives were found in higher levels only in the CTRL and ETHY (3-hexenal and 4-hexen-3-one) or in IAA and ETHY + IAA fruits (ethyl acetate). Treatment with auxin (IAA group) increased the contents of some compounds in the tomato fruits, including (Z)-2-penten-1-ol, 6-methyl-5-hepten-2-one, (E)-3-hexen-1-ol, and linalool. However, a different result was found for ETHY + IAA fruits, again suggesting that the ethylene and auxin responses overlapped within hours of treatment. Additionally, other compounds were important in distinguishing CTRL and treated fruits due to their presence in specific groups. Benzaldehyde was found only in CTRL fruits, while 2-methylbutanal was exclusive to ETHY and 1-hexanol to ETHY + IAA fruits; 1-penten-3-one and (E,E)-2,4-hexadienal were found only in CTRL and ETHY fruits.

When the CTRL fruits reached the breaker stage, the VOC profiles comprised 36 compounds; 28 of these presented abundances with significant differences (*p* < 0.05) between at least two groups. PLS-DA explained 94.9% of the total variation between samples. Component 1 (81.8%) separated the CTRL and ETHY from the IAA and ETHY + IAA groups, while component 2 (13.1%) separated the ETHY from the other groups. HCA showed that the IAA and ETHY + IAA fruits presented more similar VOC profiles, and both were more similar to the ETHY than to the CTRL fruits. Apart from 3-pentanone, which was inhibited by both exogenous treatments, fatty-acid-derived compounds were found in higher contents in treated fruits. The accumulation of hexanal, 3-hexenal, 4-hexen-3-one, (E)-2-hexenal, (E,E)-2,4-hexadienal, and (E)-2-octenal was induced by ethylene treatment, while (Z)-2-penten-1-ol, (E)-3-hexen-1-ol, and (Z)-3-hexen-1-ol were affected by auxin treatment and showed higher contents in the IAA and ETHY + IAA groups. Concerning amino-acid-derived volatiles, the accumulation of 2-methylbutanal, 2-ethylhexan-1-ol, benzaldehyde, and phenylethyl alcohol was more affected by exogenous ethylene treatment, and the (Z)-3-hexenyl and (E)-2-butenoate contents were higher in IAA and ETHY + IAA fruits. The accumulation of VOCs derived from carotenoids was separately affected by ethylene (6-methyl-5-hepten-2-one) and auxin (linalool), suggesting that each hormone regulated different sites in the geranyl diphosphate pathway. Regarding the other volatiles, heptanal, 2-propylfuran, and 2-phenylacetaldehyde were important in distinguishing ETHY fruits from the others due to their presence in this specific group. Ethyl acetate, (E)-2-pentenal, and 2-propylfuran were also important because they were exclusive to the CTRL and ETHY fruits, while 1-hexanol was exclusive to the CTRL and IAA fruits, and 1-pentanol was only found in the CTRL and ETHY + IAA fruits.

After the CTRL fruits fully ripened, their VOC profiles comprised 34 compounds, 18 of which presented abundances with significant differences (*p* < 0.05) between at least two groups. PLS-DA explained 83.2% (component 1, 61.5%; component 2, 21.7%) of the total variance between samples. The first component separated the CTRL and ETHY from the other groups, while the second separated the CTRL and IAA from ETHY and ETHY + IAA groups. As observed in the analysis with Micro-Tom fruits, HCA reinforced differences visualized by PLS-DA showing that the IAA and ETHY + IAA groups presented similar VOC profiles, as did the CTRL and ETHY groups. Fatty-acid-derived compounds had important roles in the VOC profiles of Sweet Grape tomato fruits; 3-hexenal, 2-propenal, and (E)-3-hexen-1-ol were affected by ethylene treatment, while the accumulation of propanoyl propanoate and (Z)-3-hexen-1-ol was induced by exogenous auxin. Hexanal was found in higher contents in CTRL and ETHY + IAA fruits, and 3-pentanone and 4-hexen-3-one accumulation was affected by both treatments, although these did not show higher contents in ETHY + IAA fruits. Regarding amino-acid-derived volatiles, 6-methyl-5-hepten-2-ol and 2-phenylacetaldehyde accumulation was more affected by exogenous ethylene treatment, and the 2-isobutylthiazole and 2-ethylhexan-1-ol contents were higher in IAA fruits. Additionally, other compounds were important in distinguishing the CTRL and treated fruits due to their presence in specific groups. Linalool was found only in CTRL fruits, while 2-methylbut-2-enal, (E)-2-pentenal, and benzaldehyde were exclusive to ETHY and (E,E)-2,4-hexadienal to ETHY + IAA fruits. Citral was found only in CTRL and ETHY fruits, while 1-pentanol and pentanoic acid were found only in ETHY and ETHY + IAA fruits.

### Carotenoids and Aroma-Related Gene Changes Induced by Ethylene and Auxin

**FIGURE 5:**
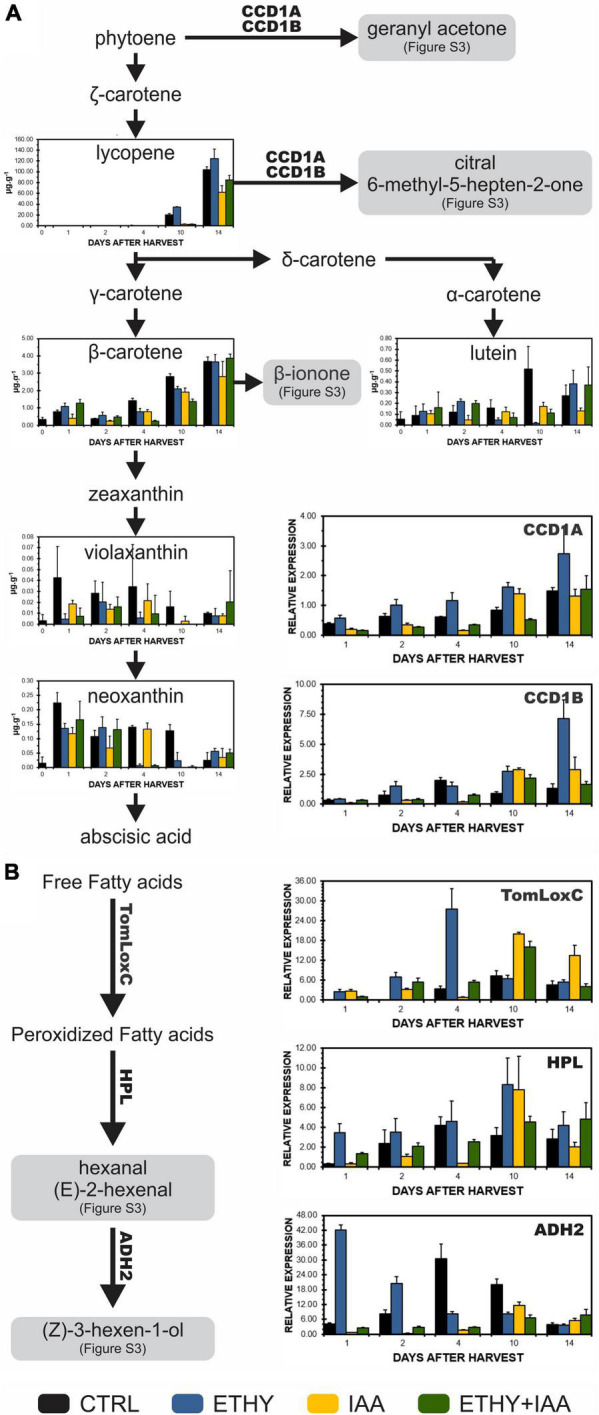
Effects of ethylene, auxin, and both treatments on carotenoid contents and genes involved in the biosynthesis of VOCs during tomato (*S. lycopersicum* L. Micro-Tom) fruit ripening. **(A)** Carotenoid content and *CCD1A* and *CCD1B* gene expression. **(B)** *TomLoxC*, *HPL*, and *ADH2* gene expression. CTRL, control group. ETHY, ethylene-treated group. IAA, indole-3-acetic acid-treated group. ETHY + IAA, group treated with both hormones. CCD, carotenoid cleavage dioxygenase. LoxC, Lipoxygenase C. HPL, hydroperoxide lyase. ADH2, alcohol dehydrogenase-2. The contents of carotenoids are given in micrograms per gram of fruit on fresh weight basis. Vertical bars represent the standard deviation of three replicates (*n* = 3).

The expression profiles of key genes involved in the biosynthesis of VOCs during Micro-Tom fruit ripening, as well as the contents of tomato fruit carotenoids, were analyzed to investigate the origin of the observed changes in tomato fruit aromas and identify the effects of exogenous auxin and ethylene treatments on the metabolic pathways of VOC precursors (Figure 5). *TomLoxC*, *HPL*, and *ADH2*, genes that code enzymes involved in the synthesis of C6 volatiles during tomato fruit ripening, were found to be upregulated by ethylene, especially at the breaker stage (04 DAH). Conversely, auxin delayed the increase in the transcriptional levels until 10 DAH. Carotenoid cleavage dioxygenase (CCD), specifically *CCD1A* and *CCD1B*, transcript levels were also downregulated by auxin and upregulated by ethylene. However, treatment with both hormones led to a similar expression pattern for IAA fruits. Lycopene contents on 10 and 14 DAH were lower in IAA and ETHY + IAA but higher in ETHY fruits, in accordance with the peel color measurements and photographs shown in Figures 1A,D. Additionally, no significant differences in the β-carotene levels were found between the groups.

### Confirmation of Direct Hormonal Effects on Micro-Tom Volatile Organic Compound Profile

**FIGURE 6:**
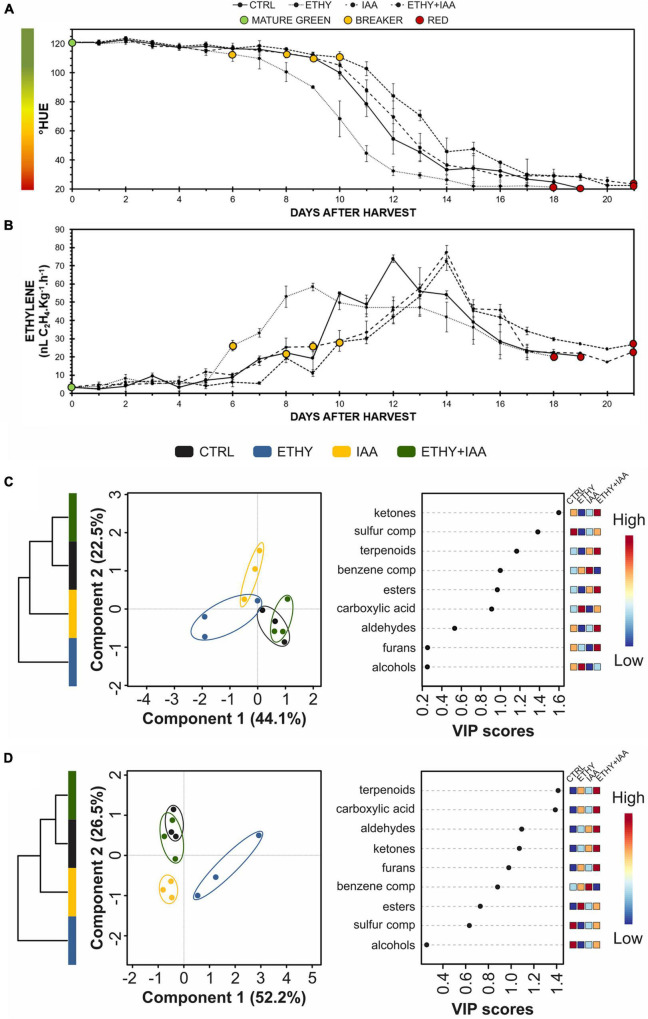
Effects of ethylene, auxin, and both treatments on fruit color, ethylene emission, and VOC contents during Micro-Tom (*S. lycopersicum* L.) fruit ripening. **(A)** Peel color expressed by hue angle. **(B)** Ethylene emission. **(C,D)** Hierarchical clustering, partial least squares discriminant analysis (PLS-DA), and variable importance in projection (VIP) of VOCs produced at breaker **(C)** and red **(D)** stages. CTRL, control group. ETHY, ethylene-treated group. IAA, indole-3-acetic acid-treated group. ETHY + IAA, group treated with both hormones. Vertical bars represent the standard deviation of three replicates (*n* = 3).

A third experiment was performed with Micro-Tom fruits to determine whether the changes observed in the VOC profiles after treatment occurred due to the direct effect of hormonal regulation or to a change in the “natural” ripening process. This new sampling directly compared the same maturation stages. The peel color shift and ethylene emission were analyzed daily (Figures 6A,B, respectively). Both parameters showed that ethylene treatment accelerated the ripening of the tomatoes, while the IAA and ETHY + IAA groups showed the opposite effect. Compared with the first Micro-Tom experiment, the CTRL fruits took on average 5 more days to reach the red stage (19 DAH), while ETHY fruits took 18 DAH and IAA and ETHY + IAA fruits took 21 DAH to fully ripen. The breaker stage was identified on the 8th DAH in CTRL fruits, the 6th day in ETHY fruits, and the 9th and 10th days in IAA and ETHY + IAA fruits.

In total, 92 VOCs were identified and confirmed at the mature green, breaker, and red stages; of these, 48 had the odoriferous note described in the literature (Supplementary Table 4). At the breaker stage (Figure 6C), VOC profiles comprised 41 compounds, 19 of which presented abundances with significant differences (*p* < 0.05) between at least two groups. PLS-DA explained 66.6% of the total variation between samples. Component 1 (44.1%) separated the CTRL and ETHY + IAA groups from the ETHY and IAA groups, while component 2 (22.5%) separated the IAA from the other groups. HCA made the differences visualized by PLS-DA clear, showing that the CTRL and ETHY + IAA groups presented similar VOC profiles, with both being more similar to the IAA group than to the ETHY group. Apart from methanethiol and 3-methylbutanal, which were inhibited by both exogenous treatments, the VOC profiles of ETHY fruits were characterized by high levels of fatty-acid-derived compounds, including hexanal, (Z)-2-hexenal, (E)-2-hexenal, and 1-octen-3-ol, besides the amino-acid derivatives 3-methyl-1-butanol and benzaldehyde. The accumulation of ethyl acetate, limonene, 3-methyl-1-pentanol, and nonanal was induced in the IAA fruits, while ETHY + IAA fruits only showed higher levels of o-guaiacol when compared with the other tomato fruits. Other fatty-acid and amino-acid derivatives had important roles in differentiating the VOC profiles. Pentanal and 1-pentanol were found in their lowest contents in IAA and ETHY + IAA fruits, in addition to 6-methyl-5-hepten-2-one, an important carotenoid-derived volatile. Normal accumulation of (E)-3-hexenal and 2-isobutylthiazole was observed in IAA fruits, with lower contents in ETHY and ETHY + IAA fruits. Ethylene and auxin treatment affected the accumulation of 2-methylfuran, 1-penten-3-one, and 2,3-pentanedione. However, these showed normal levels in ETHY + IAA fruits.

Upon reaching full ripening (Figure 6D), the VOC profiles comprised 44 compounds, 35 of which presented abundances with significant differences (*p* < 0.05) between at least two groups. PLS-DA explained 78.7% of the total variation between samples. Component 1 (52.2%) separated the ETHY from the other groups, while component 2 (26.5%) separated CTRL and ETHY + IAA fruits from those treated only with ethylene or auxin. HCA reinforced differences visualized by PLS-DA, where the CTRL and ETHY + IAA groups presented more similar VOC profiles, with both being more similar to the IAA group than to the ETHY group. Compared with treated tomatoes, CTRL fruits were better characterized by higher contents of ethyl acetate, 3-methylbutanal, 1-hexanol, and 2-isobutylthiazole. However, citral, methyl salicylate, geranyl acetone, and pentanoic acid levels were higher in ETHY and IAA fruits. Treated fruits showed altered contents of several compounds, mainly those derived from fatty acids and amino acids. The accumulation of 2,3-pentanedione, (E)-4-non-enal, and 1-octen-3-one was more affected by exogenous ethylene treatment, and the content of 3-methyl-1-pentanol was higher in IAA fruits. ETHY + IAA fruits showed higher levels of 1-penten-3-one and hexanal, two important fatty-acid-derived compounds. Benzene compounds derived from amino acids (benzaldehyde, and 1-phenylethanone) showed higher accumulation in fruits treated with ethylene and auxin, while aldehyde compounds derived from fatty acids were found in higher contents in ETHY and ETHY + IAA aromatic profiles, including (Z)-3-hexenal, heptanal, octanal, and nonanal. Additionally, methyl salicylate, 6-methyl-5-hepten-2-one, and 2-ethylfuran were also found in higher contents in ETHY and ETHY + IAA fruits.

## Discussion

Fruit ripening is a genetically coordinated process marked by significant biochemical changes in color, texture, flavor, aroma, and nutritional content that coincide with seed maturation. It is precisely regulated by a complex hormonal network. Ethylene plays critical roles in regulating fruit ripening, and the mechanisms involved in this regulatory process have been extensively studied. In contrast, however, even the exact role of auxin in ripening control remains unclear (Zhu et al., 2019). Regarding ethylene-auxin crosstalk in fruit ripening, recent studies have elucidated antagonistic effects of the two hormones. Auxin triggers ripening, acting on the transition between systems I and II of ethylene production, while ethylene acts as a positive regulator of auxin conjugation, affecting the transcript of SlGH3 genes besides the ethylene and auxin response factors (ERFs and ARFs, respectively) that regulate the biosynthesis of both hormones positively or negatively (Muday et al., 2012; McAtee et al., 2013; Kumar et al., 2014; Liu et al., 2018; Sravankumar et al., 2018; Yuan et al., 2019). In the present study, we evaluated the effects of exogenous ethylene and auxin treatments on primary ripening parameters, VOC profiles, carotenoid contents, and the expression of key genes related to regulating the metabolism of tomato aroma compounds. The color shift and ethylene emission, as primary ripening parameters, were evaluated in tomato fruits from two cultivars, Micro-Tom and Sweet Grape. Both parameters were accelerated by ethylene and delayed by auxin, while tomato fruits treated with both hormones presented intermediate responses. Previous studies reported that the tomato ripening process can be accelerated by exogenous ethylene treatment and delayed by exogenous auxin treatment (Li et al., 2016).

Tomato flavor is among the most important criteria for consumer preference (Xiao et al., 2017). The flavor of a fresh tomato is generally influenced by complex interactions of sugars, organic acids, and VOCs. During tomato ripening, several primary and secondary metabolisms are known to be controlled by regulatory mechanisms involving ethylene and auxin, including sugar and organic acid-related pathways. However, these mechanisms are not fully understood (Batista-Silva et al., 2018). VOC biosynthesis also appears to be regulated by both hormones, with most VOCs accumulating at the onset of ripening and peaking at full ripening (Klee and Giovannoni, 2011). Based on these aroma changes throughout ripening and the metabolic diversity of tomato cultivars, the VOC profiles of non-treated Micro-Tom and Sweet Grape fruits were compared at the mature green, breaker, and red stages. Multivariate analysis of the VOCs showed that the two cultivars were undiscriminated at the mature green stage, differed at the breaker stage, and clearly discriminated at the red stage. These changes observed during fruit ripening respond to hormonal regulation and several other intrinsic factors. This process promotes changes in the compound ratios and their interactions, producing changes in the tomato aroma (Klee and Giovannoni, 2011; Wang et al., 2016). Interestingly, based on their compound classes and precursors, Sweet Grape fruits presented VOC profiles more similar to the fully ripe tomato profile common to both cultivars than that of Micro-Tom.

Previous studies have shown the roles of ethylene, auxin, and other hormones (Wu et al., 2018) in regulating the VOC metabolism. However, in considering the crosstalk between the two hormones, our study unprecedentedly shows the effect of simultaneous treatment with ethylene and auxin on the tomato fruit VOC profile. Approximately 400 compounds were previously identified in tomato fruits. However, fewer than 10% are produced in sufficient quantities to be perceived by humans, being crucial in conferring the characteristic tomato aroma (Baldwin et al., 1998; Mathieu et al., 2009; Du et al., 2015). The other VOCs may provide background odors that impact the overall aroma quality (Klee and Giovannoni, 2011). Tieman et al. (2017) and Klee and Tieman (2018) described the principal contributors to the ripe tomato flavor that positively correlates with consumer preferences: 1-nitro-3-methylbutane; 1-penten-3-one; 1-octen-3-one; 2-isobutylthiazole; 2-methylbutanol; 3-methylbutanol; 2-phenylethanol; 3-pentanone; 6-methyl-5-hepten-2-ol; 6-methyl-5-hepten-2-one; benzaldehyde; heptaldehyde; nonyl aldehyde; phenylacetaldehyde; β-damascenone; citral; furaneol; geranyl acetone; guaiacol; β-ionone; isobutyl acetate; isovaleraldehyde; isovaleronitrile; isovaleric acid; methional; methyl salicylate; 1-pentanal; 1-pentanol; (E)-2-pentenal; 1-hexanal; 1-hexanol; (E)-3-hexen-1-ol; (Z)-3-hexen-1-ol; (E)-2-hexenal; (Z)-3-hexenal; (E)-2-heptenal; (E,E)-2,4-decadienal; and (Z)-4-decenal. These aroma compounds are derived from diverse precursors, including amino acids, fatty acids, and carotenoids (Klee and Tieman, 2013; Bauchet et al., 2017). In our study, Micro-Tom and Sweet Grape tomato fruits presented different VOC profiles but with similar numbers and relative areas when grouped by precursors and classes. Both cultivars had their volatile profiles changed by the hormonal treatments (ETHY, IAA, and ETHY + IAA) from the 1st day after treatment (01 DAH). VOCs derived from fatty acids [3-hexenal, (E)-2-hexenal, (Z)-3-hexen-1-ol, and others] and amino acids (2-isobutylthiazole, methyl salicylate, and others) were dominant (in number and relative area) in all the profiles of the two cultivars. Despite not representing a large chromatographic area in Sweet Grape fruits, compounds derived from carotenoids described by Tieman et al. (2017) and Klee and Tieman (2018) as positively correlated with consumer preferences (6-methyl-5-hepten-2-one and citral) were also identified in both cultivars. The multivariate analysis (PLS-DA and HCA) showed that hormonal treatments affected Micro-Tom and Sweet Grape fruits differently during the ripening process. At the breaker stage, ETHY + IAA fruits presented a VOC profile more similar to that of ETHY in Micro-Tom and that of IAA in Sweet Grape. However, the relationships between the experimental groups were the same in both cultivars when the CTRL reached the red stage. Fruits treated with both hormones presented VOC profiles more similar to that of fruits treated only with auxin, suggesting that the auxin and ethylene effects overlap.

MicroTom tomato is a mutant that expresses a truncated version of the DWARF gene, which encodes the SlCYP85A1 protein that catalyzes the oxidation of 6-deoxocastasterone to castasterone in brassinosteroid (BR) biosynthesis (Bishop et al., 1999). The mutation causes synthesis of BRs at reduced levels and results in the dwarf phenotype. The exogenous application of BRs in tomato fruits of non-defective varieties in the synthesis or response to BRs, leads to accelerated ripening, as well as the application of brassinazole, an inhibitor of BRs synthesis, delayed it (Vardhini and Rao, 2002; Zhu et al., 2015). Reports on other fruits, such as apples and pears, however, show an opposite effect of BRs on ripening, indicating different actions of this hormonal class in fruits (Ji et al., 2021). Overexpression of SlCYP90B3 in tomato fruit from Ailsa Craig cultivar resulted in higher levels of brassinolide in RED stage fruits compared to wild type, resulting in increased accumulation of carotenoids and volatile compounds characteristic of ripe fruit (Hu et al., 2020). The effect on such metabolisms is attributed to the influence of brassinolide on the expression of ACS1 and ACO1 impacting ethylene synthesis, and it has been suggested that, in tomatoes and other fruits with similar responses to higher levels, BRs would be involved in the early stages of climacteric ethylene synthesis.

Despite the low levels of BRs synthesis, the MicroTom fruit can ripen, and responds to exogenous ethylene with increased expression of several genes related to ripening, including those associated to climacteric ethylene synthesis. This seems to indicate that BRs should act in more specific aspects of ripening and that are not yet completely clear. In our results, analyzing the ethylene synthesis profiles (Figure 1) in auxin-treated groups, it is possible to see different patterns between MicroTom and Sweet Grape fruits. To confirm whether such differences are due to the lower level of BRs in MicroTom fruits, further studies with designs directed toward this end would be needed.

In our results it is observed that the global volatile profile in fruits treated with auxin and auxin plus ethylene seems to be affected differently between MicroTom and Sweet Grape fruits, mainly in the RED stage (Figure 4). While in Sweet Grape fruits the Principal Component 1 (PC1) indicates a pronounced effect of auxin in groups IAA and IAA + ETHY, in MicroTom tomatoes this second group presented an overall profile that is closer to the control group. Other variables may have contributed to this different behavior; however, it is possible to suggest that the lower levels of BRs may somehow contribute to this effect. Studies point to crosstalk between BRs and auxins in root development and hypocotyl elongation (Nemhauser et al., 2004; Kim et al., 2006), suggesting that BRs act synergistically by increasing the expression of several auxin early responsive genes. Regarding fruit ripening, there are a lack of studies that can suggest what would be the interaction between BRs and auxins. However, if BRs and auxins act synergistically in fruits as well, the results observed for MicroTom tomatoes from the IAA + ETHY group suggest that normal levels of BRs would be necessary for the effects of auxin on the synthesis of volatile compounds.

Finally, several VOCs identified in Micro-Tom and Sweet Grape and affected by the ethylene and auxin treatments are derived from fatty acids or carotenoids and contribute significantly to the characteristic tomato aroma. The carotenoid-derived volatiles are produced by the cleavage of the fruit carotenoids, mainly lycopene and β-carotene (Klee and Tieman, 2018). In our study, we analyzed these main carotenoids in addition to violaxanthin, neoxanthin, and lutein. The expression profiles of *CCD1A* and *CCD1B* genes were also analyzed during Micro-Tom fruit ripening. Hexanal, (E)-2-hexenal, (Z)-3-hexen-1-ol, and other C6 volatiles are derived from fatty acids. They are formed during the cleavage of linoleic and linolenic acids by the action of lipoxygenase C (TomLoxC) followed by oxidative cleavage catalyzed by hydroperoxide lyase (HPL) to generate hexanal and (Z)-3-hexenal, which can be isomerized to (E)-2-hexenal. The alcohol dehydrogenase 2 (ADH2) catalyzes the reduction of hexanal, (Z)-3-hexenal, and (E)-2-hexenal to 1-hexanol, (Z)-3-hexen-1-ol, and (E)-2-hexen-1-ol, respectively (Speirs et al., 1998; Chen et al., 2004; Rambla et al., 2014). *TomLoxC*, *HPL*, and *ADH2* gene expression profiles were also analyzed during Micro-Tom fruit ripening. All these genes were found to be upregulated by ethylene and downregulated by auxin, which corroborates with their VOC levels produced in these pathways, lycopene contents, and appearances (see photographs).

The results described were obtained in experiments that applied the same experimental design, as most studies used exogenous hormonal treatment to investigate fruit ripening. Since the CTRL and treated fruits were sampled and analyzed on the same DAH, we compared fruit of the same age but at a different ripening stage, potentially leading to different interpretations. Therefore, a third experiment was performed with Micro-Tom fruits to determine whether the changes observed in the VOC profiles after treatment occurred due to the direct effect of hormonal regulation or as a secondary result of the changing ripening process. This new approach allowed us to observe that the fruits treated with both hormones presented a delay in the ripening process but maintained a VOC profile similar to that the CTRL fruits. Additionally, ETHY and ETHY + IAA fruits had higher contents of the compounds described by Klee and Tieman (2018) as positively correlated with consumer preferences (methyl salicylate, 6-methyl-5-hepten-2-one, and 2-ethylfuran). This result suggests that both hormones directly regulated pathways involved in VOC biosynthesis and highlights the antagonistic effects of ethylene and auxin on aroma metabolism.

## Conclusion

Fruits of tomato cv. Micro-Tom and Sweet Grape had similar VOC profiles at the early ripening stage, with differences subsequently being observed during the ripening process. Although the two tomato fruit cultivars had distinct volatile profiles when fully ripe, Micro-Tom and Sweet Grape were similarly affected by exogenous ethylene and auxin treatments, with contrary responses to the hormones. Our results indicate that the auxin and ethylene effects on the aroma metabolism overlap and that this hormonal regulation is not cultivar-dependent. Both hormones played important roles in tomato VOC formation and regulate this metabolism differently, especially regarding carotenoid and fatty-acid-derived compounds. Moreover, the content of carotenoids and the expression of *CCD1A*, *CCD1B*, and other genes involved in forming VOCs important for the aroma of tomato fruits were also antagonistically affected by ethylene and auxin treatments. Furthermore, our results suggest that ethylene and auxin directly regulated the aroma-related pathways and VOC formation since fruits apparently at the same ripening stage had different VOC profiles.

## Data Availability Statement

The original contributions presented in the study are included in the article/Supplementary Material, further inquiries can be directed to the corresponding author.

## Author Contributions

EP, ET, and BG: conceptualization. EP: funding acquisition, project administration, supervision, and writing—review and editing. EP and LF: resources. ET, BG, VB, and EL: investigation and formal analysis. ET: visualization and writing—original draft preparation. All authors contributed to the article and approved the submitted version.

## Conflict of Interest

The authors declare that the research was conducted in the absence of any commercial or financial relationships that could be construed as a potential conflict of interest.

## Publisher’s Note

All claims expressed in this article are solely those of the authors and do not necessarily represent those of their affiliated organizations, or those of the publisher, the editors and the reviewers. Any product that may be evaluated in this article, or claim that may be made by its manufacturer, is not guaranteed or endorsed by the publisher.

## Footnotes

1
http://www.fao.org/faostat

2
http://mona.fiehnlab.ucdavis.edu/

## Supplementary Material

Supplementary Figure 1 Number and relative area of volatile organic compounds (VOCs) in tomato (*Solanum lycopersicum* L. cv. Micro-Tom) fruits after ethylene, auxin, and both treatments at 0 (mature green), 04 (breaker), and 14 (red) days after harvest (DAH). **(A)** Number and relative area of VOCs according to the compound classes. **(B)** Number and relative area of VOCs obtained from the same precursors. CTRL, control group. ETHY, ethylene-treated group. IAA, indole-3-acetic acid-treated group. ETHY + IAA, group treated with both hormones. (*n* = 3).

Supplementary Figure 2 Variable importance in projection (VIP) score plot for the most important volatile organic compound (VOC) classes identified by the PLS-DA shown in Figure 4. **(A)** VOCs identified in tomato (*S. lycopersicum* L. cv. Micro-Tom) fruits after ethylene, auxin, and both treatments at 01, 04 (breaker), and 14 (red) days after harvest (DAH). **(B)** VOCs identified in tomato (*S. lycopersicum* L. cv. Sweet Grape) fruits after ethylene, auxin, and both treatments at 01, 04 (breaker), and 08 DAH (red).

Supplementary Figure 3 Normalized area of volatile organic compounds important to tomato (*S. lycopersicum* L. cv. Micro-Tom) fruits aroma after ethylene, auxin, and both treatments throughout ripening. CTRL, control group. ETHY, ethylene-treated group. IAA, indole-3-acetic acid-treated group. ETHY + IAA, group treated with both hormones. Each value is presented as the mean, and vertical bars represent the standard deviation of three replicates (*n* = 3).

Supplementary Figure 4 Number and relative area of volatile organic compounds (VOCs) in tomato (*S. lycopersicum* L. cv. Sweet Grape) fruits after ethylene, auxin, and both treatments at 0 (mature green), 04 (breaker), and 08 (red) days after harvest (DAH). **(A)** Number and relative area of VOCs according to the compound classes. **(B)** Number and relative area of VOCs obtained from the same precursors. CTRL, control group. ETHY, ethylene-treated group. IAA, indole-3-acetic acid-treated group. ETHY + IAA, group treated with both hormones. (*n* = 3).

Supplementary Table 1 Primers used in the RT-PCR.

Supplementary Table 2 Identified volatile organic compounds in tomato (*Solanum lycopersicum* L. cv. Micro-Tom) fruit after ethylene, auxin, and both treatments throughout ripening. Data presented are *p*-values for mean comparisons between treated and control fruits (*n* = 3).

Supplementary Table 3 Identified volatile organic compounds in tomato (*S. lycopersicum* L. cv. Sweet Grape) fruit after ethylene, auxin, and both treatments throughout ripening. Data presented are *p*-values for mean comparisons between treated and control fruits (*n* = 3).

Supplementary Table 4 Identified volatile organic compounds in tomato (*S. lycopersicum* L. cv. Micro-Tom) fruits after ethylene, auxin, and both treatments at breaker and red stages. Data presented are p values for mean comparisons between treated and control fruits (*n* = 3).
