# Supplementary figures and images for: Ethylene and Auxin: Hormonal Regulation of Volatile Compound Production During Tomato (Solanum lycopersicum L.) Fruit Ripening
Source: Front Plant Sci. 2021 Dec 10;12:765897. doi: 10.3389/fpls.2021.765897 (PMC8702562; doi:10.3389/fpls.2021.765897)

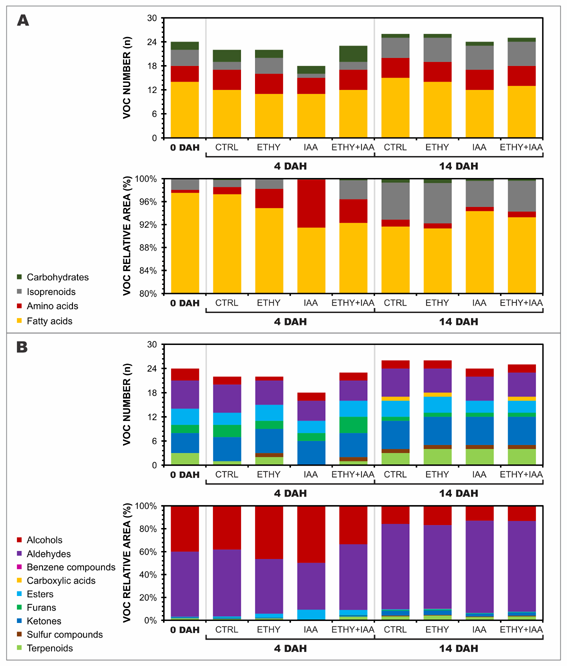

Supplement: Supplementary Figure 1 — Number and relative area of volatile organic compounds (VOCs) in tomato (Solanum lycopersicum L. cv. Micro-Tom) fruits after ethylene, auxin, and both treatments at 0 (mature green), 04 (breaker), and 14 (red) days after harvest (DAH). (A) Number and relative area of VOCs according to the compound classes. (B) Number and relative area of VOCs obtained from the same precursors. CTRL, control group. ETHY, ethylene-treated group. IAA, indole-3-acetic acid-treated group. ETHY + IAA, group treated with both hormones. (n = 3). [file Image_1.tif]

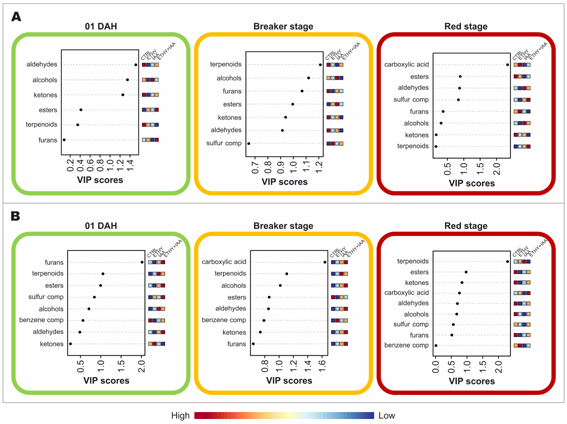

Supplement: Supplementary Figure 2 — Variable importance in projection (VIP) score plot for the most important volatile organic compound (VOC) classes identified by the PLS-DA shown in Figure 4. (A) VOCs identified in tomato (S. lycopersicum L. cv. Micro-Tom) fruits after ethylene, auxin, and both treatments at 01, 04 (breaker), and 14 (red) days after harvest (DAH). (B) VOCs identified in tomato (S. lycopersicum L. cv. Sweet Grape) fruits after ethylene, auxin, and both treatments at 01, 04 (breaker), and 08 DAH (red). [file Image_2.tif]

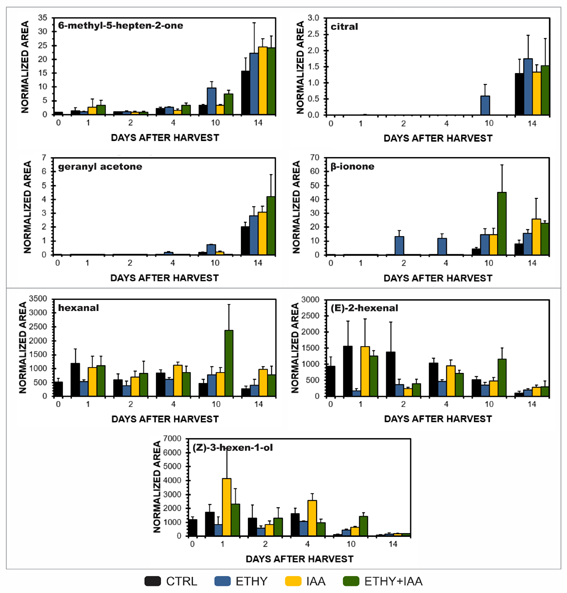

Supplement: Supplementary Figure 3 — Normalized area of volatile organic compounds important to tomato (S. lycopersicum L. cv. Micro-Tom) fruits aroma after ethylene, auxin, and both treatments throughout ripening. CTRL, control group. ETHY, ethylene-treated group. IAA, indole-3-acetic acid-treated group. ETHY + IAA, group treated with both hormones. Each value is presented as the mean, and vertical bars represent the standard deviation of three replicates (n = 3). [file Image_3.tif]

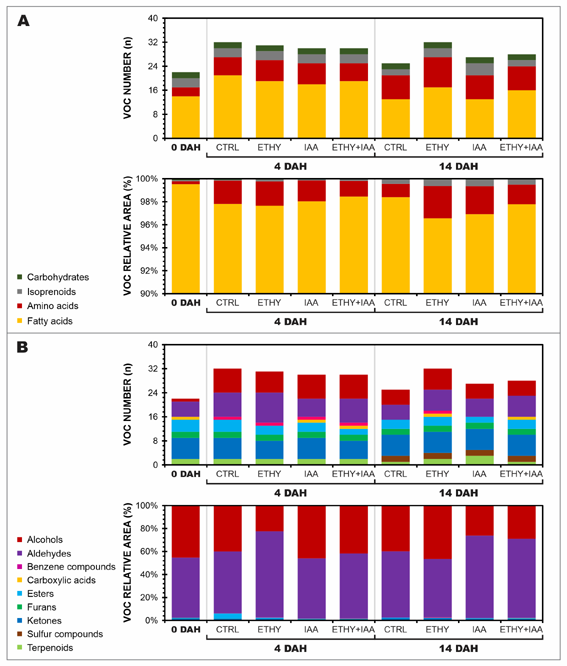

Supplement: Supplementary Figure 4 — Number and relative area of volatile organic compounds (VOCs) in tomato (S. lycopersicum L. cv. Sweet Grape) fruits after ethylene, auxin, and both treatments at 0 (mature green), 04 (breaker), and 08 (red) days after harvest (DAH). (A) Number and relative area of VOCs according to the compound classes. (B) Number and relative area of VOCs obtained from the same precursors. CTRL, control group. ETHY, ethylene-treated group. IAA, indole-3-acetic acid-treated group. ETHY + IAA, group treated with both hormones. (n = 3). [file Image_4.tif]
