# Supplementary material for: Ethylene and Auxin: Hormonal Regulation of Volatile Compound Production During Tomato (Solanum lycopersicum L.) Fruit Ripening
Source: Front Plant Sci. 2021 Dec 10;12:765897. doi: 10.3389/fpls.2021.765897 (PMC8702562; doi:10.3389/fpls.2021.765897)
Supplement: Supplementary Table 1 — Primers used in the RT-PCR. [file Table_1.docx]

Supplementary Table 1. Primers used in the RT-PCR

| GeneID | Gene name | Gene symbol | Primer | Sequence (5'-3') | Reference |
| --- | --- | --- | --- | --- | --- |
| Solyc07g025390 | Expressed unknown protein | Expressed | F | GCTAAGAACGCTGGACCTAATG | Exposito-Rodriguez et al. (2008) |
|  |  |  | R | TGGGTGTGCCTTTCTGAATG |  |
| Solyc10g049850 | TIP41-like protein | TIP41 | F | ATGGAGTTTTTGAGTCTTCTGC | Exposito-Rodriguez et al. (2008) |
|  |  |  | R | GCTGCGTTTCTGGCTTAGG |  |
| Solyc01g087250 | Carotenoid cleavage dioxygenase 1A | CCD1A | F | ATGGGGAGAAAAGAAGATGATGGA | Ilg et al. (2014) |
|  |  |  | R | ATTCAAGAACAAGCCAAACTGTGA |  |
| Solyc01g087260 | Carotenoid cleavage dioxygenase 1B | CCD1B | F | ATGGGGATGAATGAAGAAGATGGA | Ilg et al. (2014) |
|  |  |  | R | ATTCAGGAGCAAGCCAAAATGTGA |  |
| Solyc01g006540 | Lipoxygenase C | TomLoxC | F | GAGTTTGGAGTTCCAGGAGCAT | Qin et al. (2012) |
|  |  |  | R | CATCTTCGAGTGTGAGTGACTTGA |  |
| Solyc07g049690 | Hydroperoxide lyase | HPL | F | GTCCACCAGTACCAAGTCAATATGC | Bai et al. (2011) |
|  |  |  | R | GCTCCCCTTTCTTGATTTCGTAA |  |
| Solyc06g059740 | Alcohol dehydrogenase-2 | ADH2 | F | TGAGTACACCGTGGTTCATGTTG | Qin et al. (2012) |
|  |  |  | R | TCCAAGGCCTGTCGAAATTC |  |

NA: not applicable. PCR primers were designed in accordance this study specificity.
