# Supplementary material for: Ethylene and Auxin: Hormonal Regulation of Volatile Compound Production During Tomato (Solanum lycopersicum L.) Fruit Ripening
Source: Front Plant Sci. 2021 Dec 10;12:765897. doi: 10.3389/fpls.2021.765897 (PMC8702562; doi:10.3389/fpls.2021.765897)
Supplement: Supplementary Table 2 — Identified volatile organic compounds in tomato (Solanum lycopersicum L. cv. Micro-Tom) fruit after ethylene, auxin, and both treatments throughout ripening. Data presented are p-values for mean comparisons between treated and control fruits (n = 3). [file Table_2.docx]

**Supplementary Table 2.** Identified volatile organic compounds in tomato (*Solanum lycopersicum* L. cv. Micro-Tom) fruit after ethylene, auxin, and both treatments throughout ripening. Data presented are *p* values for mean comparisons between treated and control fruits (n = 3)

| ID | Volatile organic compound | RI | Functional group | Odor type | 04 DAH | | |  | 14 DAH | | |
| --- | --- | --- | --- | --- | --- | --- | --- | --- | --- | --- | --- |
|  |  |  |  |  | ETHY | IAA | ETHY+IAA |  | ETHY | IAA | ETHY+IAA |
| **Amino acids** | |  |  |  |  |  |  |  |  |  |  |
| Est.03 | methyl heptanoate | 1283 | ester | fruity | **0.01** | 0.10 | **<0.01** |  | 0.76 | 0.65 | 0.50 |
| Ket.05 | 2,2,6-trimethylcyclohexan-1-one | 1303 | ketone | thujonic | 0.36 | 0.74 | 0.64 |  | 0.42 | 0.95 | 0.55 |
| Est.04 | (Z)-3-hexenyl (E)-2-butenoate | 1361 | ester | green | **0.01** | 0.36 | 0.13 |  | 0.78 | 0.24 | 0.84 |
| Sulf.01 | 2-isobutylthiazole* | 1391 | sulfur compound | tomato | **0.01** | 0.61 | **<0.01** |  | 0.41 | 0.16 | 0.90 |
| Alc.09 | 2-ethylhexan-1-ol | 1484 | alcohol | citrus | **0.01** | **0.01** | **0.01** |  | 0.77 | 0.16 | 0.69 |
| Est.05 | methyl salicylate* | 1760 | ester | minty | 0.06 | 0.11 | 0.16 |  | 0.65 | **0.04** | 0.12 |
| Est.06 | ethyl salicylate | 1850 | ester | minty | **0.01** | **0.01** | **0.01** |  | 0.77 | 0.16 | 0.69 |
| **Carbohydrates** | |  |  |  |  |  |  |  |  |  |  |
| Fur.01 | 2-acetyl-5-methylfuran | 1081 | furan | nutty | 0.25 | **0.01** | **0.04** |  | 0.77 | 0.16 | 0.69 |
| Fur.02 | 2-pentylfuran | 1217 | furan | fruity | **0.01** | 0.40 | **<0.01** |  | 0.80 | 0.40 | 0.36 |
| Fur.03 | 2-ethylfuran | 1400 | furan | sweet | **0.05** | **0.01** | **0.02** |  | 0.77 | 0.16 | 0.69 |
| Fur.05 | 2-propylfuran | 1461 | furan | fruity | 0.06 | **0.02** | **0.04** |  | 0.77 | 0.16 | 0.69 |
| **Fatty acids** | |  |  |  |  |  |  |  |  |  |  |
| Ket.01 | 3-pentanone | <1000 | ketone | ethereal | 0.48 | 0.63 | 0.67 |  | 0.32 | 0.10 | **0.04** |
| Ket.02 | 1-penten-3-one* | 1027 | ketone | spicy | **0.03** | 0.06 | 0.07 |  | 0.82 | 0.07 | 0.10 |
| Ald.02 | hexanal* | 1084 | aldehyde | green | 0.09 | 0.58 | **0.03** |  | 0.73 | **0.02** | 0.13 |
| Ald.05 | 3-hexenal | 1151 | aldehyde | green | **<0.01** | **<0.01** | **<0.01** |  | **0.01** | **0.01** | **0.01** |
| Est.02 | propanoyl propanoate | 1173 | ester | ethereal | 0.06 | 0.61 | **0.02** |  | 0.55 | **0.02** | **0.02** |
| Ald.07 | (E)-2-hexenal* | 1219 | aldehyde | green | 0.28 | 0.14 | 0.88 |  | 0.53 | 0.87 | 0.58 |
| Ket.04 | 1-hepten-3-one | 1296 | ketone | metallic | 0.70 | 0.88 | 0.14 |  | 0.68 | 0.25 | 0.34 |
| Ket.06 | 2-heptanone | 1319 | ketone | green | 0.11 | 0.34 | 0.11 |  | 0.82 | 0.23 | 0.98 |
| Ald.08 | 2-propenal | 1352 | aldehyde | fruity | **0.01** | 0.17 | 0.48 |  | 0.39 | 0.22 | 0.63 |
| Alc.05 | (Z)-3-hexen-1-ol* | 1379 | alcohol | green | 0.11 | 0.12 | 0.22 |  | 0.73 | 0.27 | 0.49 |
| Ald.09 | nonanal | 1391 | aldehyde | aldehydic | 0.56 | 0.64 | 0.91 |  | 0.64 | 0.36 | 0.53 |
| Ald.10 | (E,E)-2,4-hexadienal | 1401 | aldehyde | green | **0.02** | **0.02** | **0.02** |  | 0.77 | 0.16 | 0.69 |
| Ald.11 | (E)-2-octenal* | 1423 | aldehyde | fatty | 0.11 | 1.00 | 0.10 |  | 0.73 | 0.22 | 0.32 |
| Alc.06 | 1-octen-3-ol | 1447 | alcohol | earthy | 0.25 | **<0.01** | **0.02** |  | 0.30 | 0.09 | 0.97 |
| Ald.12 | decanal | 1493 | aldehyde | aldehydic | **0.01** | 0.61 | 0.14 |  | 0.12 | **0.01** | **0.05** |
| Carb.01 | pentanoic acid | 1889 | carboxylic acid | cheesy | 0.25 | 0.61 | 0.14 |  | 0.21 | 0.11 | 0.41 |

**Supplementary Table 2.** (*Cont.*)

| ID | Volatile organic compound | RI | Functional group | Odor type | 04 DAH | | |  | 14 DAH | | |
| --- | --- | --- | --- | --- | --- | --- | --- | --- | --- | --- | --- |
|  |  |  |  |  | ETHY | IAA | ETHY+IAA |  | ETHY | IAA | ETHY+IAA |
| **Isoprenoids** | |  |  |  |  |  |  |  |  |  |  |
| Ket.07 | 6-methyl-5-hepten-2-one* | 1333 | ketone | citrus | **0.03** | **0.02** | **0.03** |  | 0.95 | 0.17 | 0.21 |
| Terp.01 | α-cubebene | 1456 | terpenoid | herbal | 0.25 | 0.61 | 0.08 |  | 0.77 | 0.16 | 0.69 |
| Terp.02 | linalool | 1543 | terpenoid | floral | 0.25 | 0.61 | 0.14 |  | 0.77 | 0.16 | 0.69 |
| Terp.03 | linalyl butanoate | 1548 | terpenoid | floral | 0.25 | 0.61 | 0.14 |  | 0.77 | 0.16 | 0.69 |
| Terp.05 | β-cyclocitral | 1601 | terpenoid | tropical | 1.00 | **0.05** | **0.05** |  | 0.68 | 0.14 | 0.59 |
| Terp.06 | citral* | 1724 | terpenoid | citrus | 0.25 | 0.61 | 0.14 |  | 0.88 | **0.03** | 0.07 |
| Terp.07 | photocitral A | 1729 | terpenoid | herbal | 0.25 | 0.61 | 0.14 |  | 0.10 | **<0.01** | **0.02** |
| Ket.09 | geranyl acetone* | 1850 | ketone | floral | **0.04** | 0.61 | 0.14 |  | 0.84 | 0.09 | 0.61 |
| Terp.08 | β-ionone* | 1924 | terpenoid | floral | **0.03** | 0.61 | 0.14 |  | 0.36 | 0.61 | 0.36 |

ID: Volatile compound identification. RI: Retention index. relative to n-alkanes (C7-C30) on the SupelcoWax capillary column. ETHY: *p* value for T-test between control and ethylene treated fruits. IAA: *p* value for T-test between control and auxin treated fruits. ETHY+IAA: *p* value for T-test between control and ethylene-auxin treated fruits. DAH: Days after harvest. *Compound confirmed by mass spectrum comparison with external standard. Values in bold letters show significant differences (*p* < 0.05) between the control and treated fruits.
