# Supplementary material for: Ethylene and Auxin: Hormonal Regulation of Volatile Compound Production During Tomato (Solanum lycopersicum L.) Fruit Ripening
Source: Front Plant Sci. 2021 Dec 10;12:765897. doi: 10.3389/fpls.2021.765897 (PMC8702562; doi:10.3389/fpls.2021.765897)
Supplement: Supplementary Table 3 — Identified volatile organic compounds in tomato (S. lycopersicum L. cv. Sweet Grape) fruit after ethylene, auxin, and both treatments throughout ripening. Data presented are p-values for mean comparisons between treated and control fruits (n = 3). [file Table_3.docx]

**Supplementary Table 3.** Identified volatile organic compounds in tomato (*Solanum lycopersicum* L. cv. Sweet Grape) fruit after ethylene, auxin, and both treatments throughout ripening. Data presented are *p* values for mean comparisons between treated and control fruits (n = 3)

| ID | Volatile organic compound | RI | Functional group | Odor type | 04 DAH | | |  | 08 DAH | | |
| --- | --- | --- | --- | --- | --- | --- | --- | --- | --- | --- | --- |
|  |  |  |  |  | ETHY | IAA | ETHY+IAA |  | ETHY | IAA | ETHY+IAA |
| **Amino acids** | |  |  |  |  |  |  |  |  |  |  |
| Ald.01 | 2-methylbutanal | <1000 | aldehyde | chocolate | 0.81 | 0.83 | 0.46 |  | 0.64 | 0.93 | 0.70 |
| Ald.03 | 2-methylbut-2-enal | 1095 | aldehyde | green | 0.26 | 0.74 | 0.45 |  | 0.47 | **0.03** | 0.06 |
| Est.03 | methyl heptanoate | 1283 | ester | fruity | 0.06 | 0.23 | **0.01** |  | **0.05** | **0.02** | **0.03** |
| Est.04 | (Z)-3-hexenyl (E)-2-butenoate | 1361 | ester | green | **<0.01** | 0.09 | 0.26 |  | 0.61 | 0.98 | 0.60 |
| Sulf.01 | 2-isobutylthiazole* | 1391 | sulfur compound | tomato | 0.26 | 0.74 | 0.45 |  | **0.05** | 0.93 | 0.12 |
| Alc.08 | 6-methyl-5-hepten-2-ol | 1458 | alcohol | green | 0.26 | 0.74 | 0.45 |  | 0.76 | 0.09 | 0.06 |
| Alc.09 | 2-ethylhexan-1-ol | 1484 | alcohol | citrus | 0.69 | 0.82 | 0.77 |  | 0.84 | 0.70 | 0.24 |
| Benz.04 | benzaldehyde | 1506 | benzene compound | fruity | **0.01** | 0.93 | 0.95 |  | **0.01** | 0.93 | 0.70 |
| Ald.13 | 2-phenylacetaldehyde | 1622 | aldehyde | green | **<0.01** | **0.01** | **0.02** |  | 0.97 | 0.25 | 0.86 |
| Sulf.02 | 2-thiolpropionic acid | 1697 | sulfur compound | sulfurous | 0.26 | 0.74 | 0.45 |  | 0.30 | 0.33 | 0.18 |
| Est.05 | methyl salicylate* | 1760 | ester | minty | 0.26 | 0.74 | 0.45 |  | 0.64 | 0.93 | 0.70 |
| Alc.10 | 2-phenylethanol * | 1888 | alcohol | floral | **0.01** | 0.49 | **0.03** |  | 0.64 | 0.96 | 0.92 |
|  | **Carbohydrates** |  |  |  |  |  |  |  |  |  |  |
| Fur.03 | 2-ethylfuran | 1400 | furan | sweet | **0.02** | **0.01** | 0.08 |  | 0.28 | 0.41 | 0.09 |
| Fur.04 | 2-pentanoylfuran | 1458 | furan | caramelly | 0.26 | 0.74 | 0.45 |  | 0.40 | 0.16 | 0.81 |
| Fur.05 | 2-propylfuran | 1461 | furan | fruity | 0.22 | **0.01** | **0.02** |  | 0.64 | 0.93 | 0.70 |
| **Fatty acids** | |  |  |  |  |  |  |  |  |  |  |
| Ket.01 | 3-pentanone | <1000 | ketone | ethereal | 0.07 | 0.19 | 0.44 |  | 0.13 | 0.35 | 0.84 |
| Est.01 | ethyl acetate | <1000 | ester | fruity | 0.06 | **0.03** | **0.03** |  | 0.64 | 0.93 | 0.70 |
| Ket.02 | 1-penten-3-one* | 1027 | ketone | spicy | 0.12 | **0.05** | **0.01** |  | 0.29 | 0.11 | 0.12 |
| Ald.02 | hexanal* | 1084 | aldehyde | green | **0.02** | 0.70 | 0.71 |  | 0.24 | 0.11 | 0.21 |
| Ald.04 | (E)-2-pentenal | 1130 | aldehyde | green | 0.07 | **0.01** | **0.01** |  | 0.14 | 0.93 | 0.70 |
| Ald.05 | 3-hexenal | 1151 | aldehyde | green | **0.01** | **0.04** | 0.69 |  | 0.63 | 0.90 | 0.69 |
| Ket.03 | 4-hexen-3-one | 1152 | ketone | acidic | 0.09 | **0.05** | 0.81 |  | 0.88 | 0.58 | 0.67 |
| Est.02 | propanoyl propanoate | 1173 | ester | ethereal | 0.06 | 0.57 | 0.72 |  | 0.65 | 0.39 | 0.67 |
| Ald.06 | heptanal | 1185 | aldehyde | green | 0.12 | 0.74 | 0.45 |  | 0.64 | 0.93 | 0.70 |
| Ald.07 | (E)-2-hexenal* | 1219 | aldehyde | green | 0.11 | 0.42 | 0.19 |  | 0.43 | 0.64 | 0.30 |
| Alc.01 | 1-pentanol* | 1246 | alcohol | fermented | **0.01** | **0.01** | 0.39 |  | **0.02** | 0.93 | **0.03** |
| Ket.04 | 1-hepten-3-one | 1296 | ketone | metallic | 0.28 | 0.23 | 0.14 |  | 0.56 | 0.46 | 0.51 |

**Supplementary Table 3.** (*Cont.*)

| ID | Volatile organic compound | RI | Functional group | Odor type | 04 DAH | | |  | 08 DAH | | |
| --- | --- | --- | --- | --- | --- | --- | --- | --- | --- | --- | --- |
|  |  |  |  |  | ETHY | IAA | ETHY+IAA |  | ETHY | IAA | ETHY+IAA |
| **Fatty acids** | |  |  |  |  |  |  |  |  |  |  |
| Alc.02 | (Z)-2-penten-1-ol | 1314 | alcohol | green | 0.14 | 0.70 | 0.52 |  | 0.64 | 0.93 | 0.70 |
| Ket.06 | 2-heptanone | 1319 | ketone | green | **0.05** | 0.77 | 0.59 |  | 0.66 | 0.97 | 0.70 |
| Alc.03 | 1-hexanol* | 1348 | alcohol | herbal | **<0.01** | 0.29 | **<0.01** |  | **<0.01** | 0.93 | 0.70 |
| Ald.08 | 2-propenal | 1352 | aldehyde | fruity | 0.44 | 0.24 | 0.10 |  | 0.96 | 0.73 | 0.62 |
| Alc.04 | (E)-3-hexen-1-ol | 1357 | alcohol | green | **<0.01** | 0.07 | 0.12 |  | 0.69 | 0.99 | 0.47 |
| Alc.05 | (Z)-3-hexen-1-ol* | 1379 | alcohol | green | **0.02** | 0.27 | 0.14 |  | 0.94 | 0.41 | 1.00 |
| Ald.10 | (E,E)-2,4-hexadienal | 1401 | aldehyde | green | 0.07 | 0.06 | 0.72 |  | 0.64 | 0.93 | 0.26 |
| Ket.08 | 1-octen-3-one | 1421 | ketone | earthy | 0.64 | 0.92 | 0.70 |  | 0.92 | 0.38 | 0.48 |
| Ald.11 | (E)-2-octenal* | 1423 | aldehyde | fatty | 0.64 | **0.01** | **0.01** |  | 0.64 | 0.93 | 0.70 |
| Alc.06 | 1-octen-3-ol | 1447 | alcohol | earthy | 0.22 | 0.50 | 0.30 |  | 0.64 | 0.93 | 0.70 |
| Alc.07 | 1-heptanol | 1448 | alcohol | green | **<0.01** | **0.01** | **0.01** |  | 0.64 | 0.93 | 0.70 |
| Carb.01 | pentanoic acid | 1889 | carboxylic acid | cheesy | 0.26 | **0.02** | **<0.01** |  | **0.03** | 0.93 | **0.01** |
| **Isoprenoids** | |  |  |  |  |  |  |  |  |  |  |
| Ket.07 | 6-methyl-5-hepten-2-one* | 1333 | ketone | citrus | **0.04** | 0.26 | **0.03** |  | 0.88 | 0.56 | 0.28 |
| Terp.02 | linalool | 1543 | terpenoid | floral | 0.11 | 0.25 | 0.40 |  | 0.07 | 0.07 | 0.07 |
| Terp.04 | o-guaiacol* | 1597 | terpenoid | woody | 0.11 | 0.29 | 0.15 |  | 0.15 | 0.76 | 0.15 |
| Terp.06 | citral* | 1724 | terpenoid | citrus | 0.26 | 0.74 | 0.45 |  | 0.64 | 0.06 | 0.06 |

ID: Volatile compound identification. RI: Retention index. relative to n-alkanes (C7-C30) on the SupelcoWax capillary column. ETHY: *p* value for T-test between control and ethylene treated fruits. IAA: *p* value for T-test between control and auxin treated fruits. ETHY+IAA: *p* value for T-test between control and ethylene-auxin treated fruits. DAH: Days after harvest. *Compound confirmed by mass spectrum comparison with external standard. Values in bold letters show significant differences (*p* < 0.05) between the control and treated fruits.
