# Supplementary material for: Ethylene and Auxin: Hormonal Regulation of Volatile Compound Production During Tomato (Solanum lycopersicum L.) Fruit Ripening
Source: Front Plant Sci. 2021 Dec 10;12:765897. doi: 10.3389/fpls.2021.765897 (PMC8702562; doi:10.3389/fpls.2021.765897)
Supplement: Supplementary Table 4 — Identified volatile organic compounds in tomato (S. lycopersicum L. cv. Micro-Tom) fruits after ethylene, auxin, and both treatments at breaker and red stages. Data presented are p values for mean comparisons between treated and control fruits (n = 3). [file Table_4.docx]

**Supplementary Table 4.** Identified volatile organic compounds in tomato (*Solanum lycopersicum* L. cv. Micro-Tom) fruits after ethylene, auxin, and both treatments at breaker and red stages. Data presented are p values for mean comparisons between treated and control fruits (n = 3)

| ID | Volatile organic compound | RI | Functional group | Odor type | Breaker | | |  | Red | | |
| --- | --- | --- | --- | --- | --- | --- | --- | --- | --- | --- | --- |
|  |  |  |  |  | ETHY | IAA | ETHY+IAA |  | ETHY | IAA | ETHY+IAA |
| **Amino acids** | |  |  |  |  |  |  |  |  |  |  |
| Sulf.01 | methanethiol | <900 | sulfur compound | sulfurous | 0.40 | 0.31 | 0.39 |  | 0.15 | 0.17 | 0.46 |
| Ald.02 | 3-methylbutanal | <900 | aldehyde | aldehydic | **0.05** | 0.10 | 0.11 |  | **0.04** | 0.13 | **0.05** |
| Ket.03 | 2-butyl acetate | 969 | ketone | fruity | **0.03** | 0.30 | 0.26 |  | **0.01** | **0.01** | **0.02** |
| Ket.06 | 3,3,6-Trimethylhepta-1,5-dien-4-one | 1080 | ketone | herbal | **0.05** | **0.01** | 0.72 |  | 0.06 | 0.33 | 0.27 |
| Alc.01 | 3-methyl-1-butanol | 1213 | alcohol | fermented | **0.05** | 0.09 | **0.05** |  | 0.17 | 0.24 | **0.01** |
| Alc.03 | 3-methyl-1-pentanol | 1324 | alcohol | fruity | **0.03** | 0.15 | 0.18 |  | 0.06 | **0.02** | 0.13 |
| Sulf.02 | 2-isobutylthiazole* | 1391 | sulfur compound | tomato | **<0.01** | **0.01** | **0.02** |  | **<0.01** | **0.01** | **0.03** |
| Benz.06 | benzaldehyde | 1506 | benzene compound | fruity | **0.05** | 0.09 | 0.26 |  | 0.09 | **0.03** | 0.06 |
| Benz.07 | 1-phenylethanone | 1632 | benzene compound | floral | 0.50 | 0.13 | 0.83 |  | **0.05** | **<0.01** | 0.15 |
| Carb.03 | 3-methylbutanoic acid | 1657 | carboxylic acid | cheesy | 0.18 | 0.58 | 0.97 |  | 0.12 | 0.65 | 0.08 |
| Est.04 | methyl salicylate* | 1760 | ester | minty | 0.18 | 0.18 | 0.17 |  | 0.13 | 0.60 | 0.45 |
| Alc.06 | 2-phenylethanol* | 1888 | alcohol | floral | **0.03** | **<0.01** | 0.26 |  | 0.22 | 0.18 | 0.14 |
| **Carbohydrates** | |  |  |  |  |  |  |  |  |  |  |
| Fur.01 | 2-methylfuran | <900 | furan | chocolate | 0.33 | 0.10 | 0.41 |  | 0.29 | 0.13 | 0.95 |
| Fur.02 | 2-ethylfuran | 1400 | furan | sweet | 0.33 | 0.97 | 0.80 |  | 0.66 | 0.16 | 0.35 |
| Fur.03 | 2-propylfuran | 1442 | furan | fruity | **0.03** | 0.30 | 0.26 |  | 0.06 | 0.42 | 0.13 |
| **Fatty acids** | |  |  |  |  |  |  |  |  |  |  |
| Est.01 | ethyl ether | <900 | ether | ethereal | 0.18 | 0.33 | 0.35 |  | 0.15 | 0.22 | 0.32 |
| Ald.01 | Propanal | <900 | aldehyde | ethereal | **<0.01** | 0.96 | **0.03** |  | 0.70 | 0.79 | 0.16 |
| Ket.01 | propan-2-one | <900 | ketone | solvent | 0.29 | 0.99 | 0.76 |  | **0.03** | 0.71 | 0.29 |
| Ket.02 | methyl acetate | <900 | ketone | ethereal | **0.05** | 0.61 | 0.76 |  | **0.04** | **0.04** | 0.08 |
| Est.02 | ethyl acetate | <900 | ester | fruity | 0.24 | 0.58 | 0.67 |  | 0.34 | 0.34 | 0.39 |
| Ald.03 | pentanal* | 958 | aldehyde | fermented | **0.03** | 0.16 | 0.37 |  | 0.27 | 0.37 | **0.01** |
| Ket.04 | 1-penten-3-one | 1027 | ketone | spicy | 0.11 | 0.06 | 0.89 |  | 0.15 | 0.55 | 0.68 |
| Ket.05 | 2,3-pentanedione | 1044 | ketone | buttery | **0.01** | **0.01** | 0.82 |  | 0.55 | **0.01** | 0.39 |
| Carb.01 | methyl pentanoate | 1072 | carboxylic acid | fruity | **0.03** | 0.30 | 0.08 |  | 0.06 | 0.42 | 0.13 |
| Ald.04 | hexanal* | 1084 | aldehyde | green | 0.10 | 0.79 | 0.49 |  | 0.14 | 0.28 | 0.50 |
| Ald.05 | (E)-3-hexenal | 1120 | aldehyde | green | **0.03** | 0.50 | 0.09 |  | 0.72 | 0.25 | 0.38 |
| Ald.06 | (Z)-3-hexenal* | 1126 | aldehyde | green | **0.02** | 0.14 | 0.13 |  | 0.78 | 0.30 | 0.64 |

**Supplementary Table 4.** (*Cont.*)

| ID | Volatile organic compound | RI | Functional group | Odor type | Breaker | | |  | Red | | |
| --- | --- | --- | --- | --- | --- | --- | --- | --- | --- | --- | --- |
|  |  |  |  |  | ETHY | IAA | ETHY+IAA |  | ETHY | IAA | ETHY+IAA |
| **Fatty acids** | |  |  |  |  |  |  |  |  |  |  |
| Ket.07 | 4-hexen-3-one | 1152 | ketone | acidic | 0.42 | 0.58 | 0.89 |  | 0.06 | 0.42 | 0.13 |
| Est.03 | propanoyl propanoate | 1173 | ester | ethereal | **0.03** | 0.30 | 0.26 |  | **0.03** | 0.42 | **0.01** |
| Ald.07 | heptanal | 1185 | aldehyde | green | 0.39 | 0.18 | 0.22 |  | 0.77 | 0.74 | 0.41 |
| Ald.08 | (Z)-2-hexenal | 1200 | aldehyde | green | 0.15 | 0.74 | 0.73 |  | **0.05** | 0.19 | 0.13 |
| Ald.09 | (E)-2-hexenal* | 1219 | aldehyde | green | 0.65 | 0.41 | 0.43 |  | 0.24 | 0.70 | 0.34 |
| Alc.02 | 1-pentanol* | 1246 | alcohol | fermented | **0.03** | 0.16 | 0.42 |  | 0.08 | 0.85 | 0.62 |
| Ald.10 | octanal | 1268 | aldehyde | aldehydic | 0.08 | 0.82 | 0.19 |  | **0.03** | 0.47 | 0.32 |
| Alc.04 | 1-hexanol* | 1348 | alcohol | herbal | **0.02** | 0.30 | 0.26 |  | **0.02** | **0.03** | **0.05** |
| Ald.11 | nonanal | 1391 | aldehyde | aldehydic | **0.04** | **0.01** | 0.06 |  | **0.01** | **0.01** | **0.04** |
| Ald.12 | (E)-4-nonenal | 1416 | aldehyde | fruity | **0.03** | 0.30 | 0.26 |  | **0.03** | **0.01** | 0.17 |
| Ket.09 | 1-octen-3-one | 1421 | ketone | earthy | 0.21 | **0.02** | 0.33 |  | 0.94 | 0.86 | 0.06 |
| Ald.13 | (E)-2-octenal* | 1423 | aldehyde | fatty | **<0.01** | 0.30 | 0.11 |  | 0.11 | **<0.01** | **0.03** |
| Carb.02 | acetic acid | 1439 | carboxylic acid | acidic | 0.08 | 0.51 | 0.57 |  | **<0.01** | **0.03** | **0.02** |
| Alc.05 | 1-octen-3-ol | 1447 | alcohol | earthy | **0.01** | 0.28 | 0.46 |  | **0.01** | **0.01** | 0.29 |
| Carb.04 | pentanoic acid | 1889 | carboxylic acid | cheesy | 0.29 | 0.13 | **0.03** |  | 0.24 | 0.39 | 0.57 |
| **Isoprenoids** | |  |  |  |  |  |  |  |  |  |  |
| Terp.01 | α-pinene | 1005 | terpenoid | herbal | **0.03** | 0.30 | 0.26 |  | 0.06 | 0.42 | 0.13 |
| Terp.02 | limonene | 1161 | terpenoid | citrus | **0.01** | 0.19 | 0.53 |  | **0.02** | 0.42 | 0.13 |
| Ket.08 | 6-methyl-5-hepten-2-one* | 1333 | ketone | citrus | 0.16 | 0.10 | 0.66 |  | 0.22 | 0.55 | **0.03** |
| Terp.03 | o-guaiacol* | 1597 | terpenoid | woody | 0.12 | 0.28 | **0.04** |  | 0.06 | 0.42 | 0.07 |
| Terp.04 | citral* | 1724 | terpenoid | citrus | **0.03** | 0.30 | 0.26 |  | **0.05** | 0.33 | **0.02** |
| Ket.10 | geranyl acetone* | 1850 | ketone | floral | **0.03** | 0.30 | 0.26 |  | 0.44 | 0.18 | 0.15 |

ID: Volatile compound identification. RI: Retention index. relative to n-alkanes (C7-C30) on the SupelcoWax capillary column. ETHY: *p* value for T-test between control and ethylene treated fruits. IAA: *p* value for T-test between control and auxin treated fruits. ETHY+IAA: *p* value for T-test between control and ethylene-auxin treated fruits. *Compound confirmed by mass spectrum comparison with external standard. Values in bold letters show significant differences (*p* < 0.05) between the control and treated fruits.
